# ROS‐Activated Nanohydrogel Scaffolds with Multi‐Factors Controlled Release for Targeted Dual‐Lineage Repair of Osteochondral Defects

**DOI:** 10.1002/advs.202412410

**Published:** 2025-03-29

**Authors:** Xiuhui Wang, Shunli Wu, Ruiyang Li, Huijian Yang, Yue Sun, Zijie Cao, Xiao Chen, Yan Hu, Hao Zhang, Zhen Geng, Long Bai, Zhongmin Shi, Ke Xu, Hongbo Tan, Jiacan Su

**Affiliations:** ^1^ Institute of Translational Medicine Shanghai University Shanghai 200444 China; ^2^ Organoid Research Center Shanghai University Shanghai 200444 China; ^3^ National Center for Translational Medicine (Shanghai) SHU Branch Shanghai University Shanghai 200444 China; ^4^ Department of Orthopedics, Xinhua Hospital Shanghai Jiao Tong University School of Medicine Shanghai 200092 China; ^5^ Department of Clinical Laboratory Shanghai Zhongye Hospital Shanghai 200941 China; ^6^ Department of Orthopaedics People's Liberation Army Joint Logistic Support Force 920th Hospital Kunming 650118 China; ^7^ National Center for Orthopaedics Department of Orthopedic Surgery Shanghai Sixth People's Hospital Shanghai 200233 China

**Keywords:** controlled release, dual‐lineage differentiation, nanohydrogel scaffolds, osteochondral regeneration, ROS‐activated

## Abstract

Achieving self‐healing for osteochondral defects caused by trauma, aging, or disease remains a significant challenge in clinical practice. It is an effective therapeutic strategy to construct gradient‐biomimetic biomaterials that replicate the hierarchical structure and complex microenvironment of osteochondral tissues for dual‐lineage regeneration of both cartilage and subchondral bone. Herein, ROS‐activated nanohydrogels composite bilayer scaffolds with multi‐factors controlled release are rationally designed using the combination of 3D printing and gelatin placeholder methods. The resulting nanohydrogel scaffolds exhibit micro‐nano interconnected porous bilayer structure and soft‐hard complex mechanical strength for facilitating 3D culture of BMSCs in vitro. More importantly, multi‐stage continuous responses of anti‐inflammation, chondrogenesis and osteogenesis, are effectively induced via the sequential release of multi‐factors, including diclofenac sodium (DS), kartogenin (KGN) and bone morphogenetic protein 2 (BMP‐2), from ROS‐activated nanohydrogel scaffolds, thereby improved dual‐lineage regeneration of cartilage and subchondral bone tissue in the osteochondral defect model of SD rats. These findings suggest that ROS‐activated nanohydrogel scaffolds with such specific soft‐hard bilayer structure and sequential delivery of functional factors, provides a promising strategy in dual‐lineage regeneration of osteochondral defects.

## Graphical abstract

ROS‐activated nanohydrogels composite bilayer scaffolds are rationally designed for targted dual‐lineage repair of osteochondral defects. The as‐prepared nanohydrogel scaffolds provided 3D culture and induced dual‐lineage differentiation of BMSCs in vitro, efficiently inhibited early‐stage inflammation by rapidly releasing DS and improved cartilage and subchondral bone tissue regeneration by controlled release of KGN and BMP2 via ROS stimulation in osteochondral defects model of SD rats in vivo.

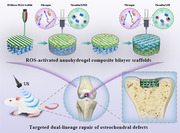

## 1 Introduction

Osteochondral defects, caused by trauma, aging or disease, are a complex pathological condition involving lesions of both the articular hyaline cartilage and subchondral bone.^[^ ^1^ ^]^ From cartilage to subchondral bone, its chemical composition, mechanical strength and biochemical microenvironment are changed.^[^ ^2^ ^]^ Although invasive surgeries including microfracture, autograft/allograft transplantation, and joint replacement could improve joint function to some extent,^[^ ^3^ ^]^ they still have limitations such as donor shortage and immune rejection.^[^ ^4^ ^]^ Hence, it is necessary to rationally design gradient bionic biomaterials to mimic the osteochondral structure and reconstruct regeneration microenvironment, thereby enabling the repair of osteochondral defect.^[^ ^5^ ^]^

In recent years, tissue engineering scaffolds with bi‐layer structure and multi‐functional composition have been wildly explored for osteochondral defects,^[^ ^6^ ^]^ which could simulate the structure of the entire osteochondral unit, further facilitate the regeneration repair of both the cartilaginous and bony regions.^[^ ^7^ ^]^ Wu et al. fabricated a novel 3D‐printed lithium‐calcium‐silicate crystal bioceramics bilayer scaffolds with such specific ionic combination that possessed dual bioactivities to meet the regeneration requirements of both cartilage and subchondral bone.^[^ ^8^ ^]^ These 3D‐printed scaffolds with different composition (PCL, PLGA, HAp, etc) and multilevel hierarchical structure offer the distinct mechanical biological requirement for various of tissues.^[^ ^9^ ^]^ Despite promising advances, these scaffolds still face challenges in providing a truly 3D growth microenvironment and facilitating the multi‐stage continuous biological responses.^[^ ^10^ ^]^ Meanwhile, cells predominantly adhere on the wall surface of porous scaffolds, maintaining a 2D culture state rather than achieving truly 3D cultivation.^[^ ^11^ ^]^ Unlike traditional scaffolds, biohydrogels (GelMA, chitosan, PEG, etc.) provide a biomimetic microenvironment that more closely mimics native extracellular matrix (ECM), supporting 3D cell cultivation and enhancing tissue regeneration.^[^ ^12^ ^]^ Herein, we creatively proposed a soft‐hard concept to construct soft matrix‐based hydrogel composite hard matrix‐based scaffolds.^[^ ^13^ ^]^ This novel nanohydrogel scaffolds not only offer spatial mechanical support through 3D‐printed complex structure but also recreate 3D matrix microenvironments with matrix‐mimic hydrogels, thereby promoting osteochondral healing.^[^ ^14^ ^]^

In addition, repair of osteochondral defect is a multi‐stage continuous physiological process,^[^ ^15^ ^]^ including hemostasis, inflammation, proliferation and maturation.^[^ ^16^ ^]^ While current tissue‐engineered strategies only focus on regulating a single regeneration stage,^[^ ^17^ ^]^ but disregarding their integrity and continuity of different repair stages.^[^ ^18^ ^]^ Sequential drug delivery strategies based on the theory of the healing cascade are expected to achieve multi‐stage synergistic therapy of osteochondral defects.^[^ ^19^ ^]^ Li et al. designed an injectable hydrogel (SA/BG‐SACM‐PLGAPFD) with multi‐layer structure to realize sequential delivery of the bioactive molecules for fulfilling the bioactivity requirement of each wound healing stage.^[^ ^20^ ^]^ Little et al. prepared a hybrid calcium phosphate/alginate scaffold to achieve programmed sequential delivery of particular growth factors, including PDGF and BMP‐2, for improving the upregulation of several regenerative stages in bone tissue engineering.^[^ ^21^ ^]^ Dai et al. Developed a 3D bioprinting dual‐factor releasing and gradient‐structured scaffolds for anisotropic cartilage regeneration.^[^ ^22^ ^]^ More importantly, we previously developed ROS‐activated nanohydrogels that enable the controlled release of KGN and employ ultrasonic stimuli to trigger ROS production, thereby achieving the repair of irregular cartilage defects.^[^ ^23^ ^]^ Herein, all strategies including soft‐hard gradient structure, bionic matrix components and multi‐factors controlled release the construction of hydrogel scaffolds will facilitate multilevel tissue regeneration.^[^ ^24^ ^]^

**Scheme 1:**
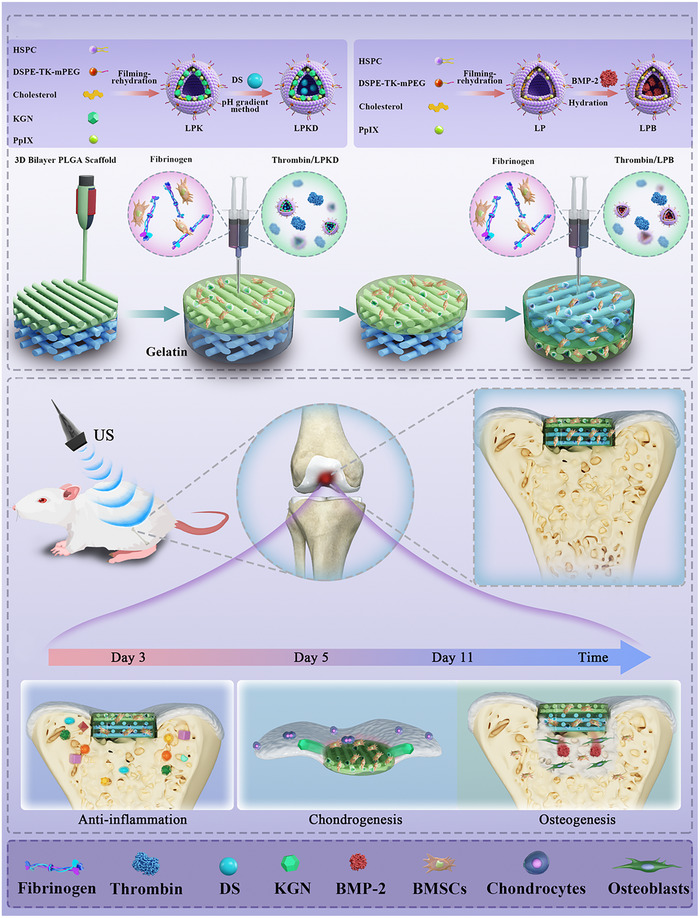
Schematic illustration of rational design of multi‐factors loaded nanohydrogels composite bilayer scaffolds with sequential regulation for osteochondral regeneration.

Taken together, a novel multi‐factors loaded nanohydrogel composite bilayer scaffold with ROS‐controlled release is rationally designed to handle each stage of osteochondral regeneration (**Scheme** **1**). In which, DS, KGN and BMP‐2 were individually loaded into ROS responsive liposomes, and then mixed with the precursor of fibrin hydrogels (FT) using a dual‐syringe system. This mixture was injected into the upper layer (PLGA‐FT‐LPKD) and lower layer (PLGA‐FT‐LPB) of 3D‐printed bilayer PLGA scaffold using a gelatin placeholder method, respectively. As a result, enzymatic reaction occurred within the bilayer scaffold to construct multi‐factors loaded nanohydrogels composite bilayer scaffolds. Subsequently, the sequential release of multi‐factors under ultrasonic stimulation and 3D culture of BMSCs within hydrogels composite bilayer scaffolds induced stepwise responses including anti‐inflammation, chondrogenesis and osteogeneis, thereby improved osteochondral repair in vitro and in vivo.

## 2 Results and Discussion

### 2.1 Synthesis and Characterization of ROS‐Responsive Nanosystem with Multi‐Factors Loading

**Figure 1:**
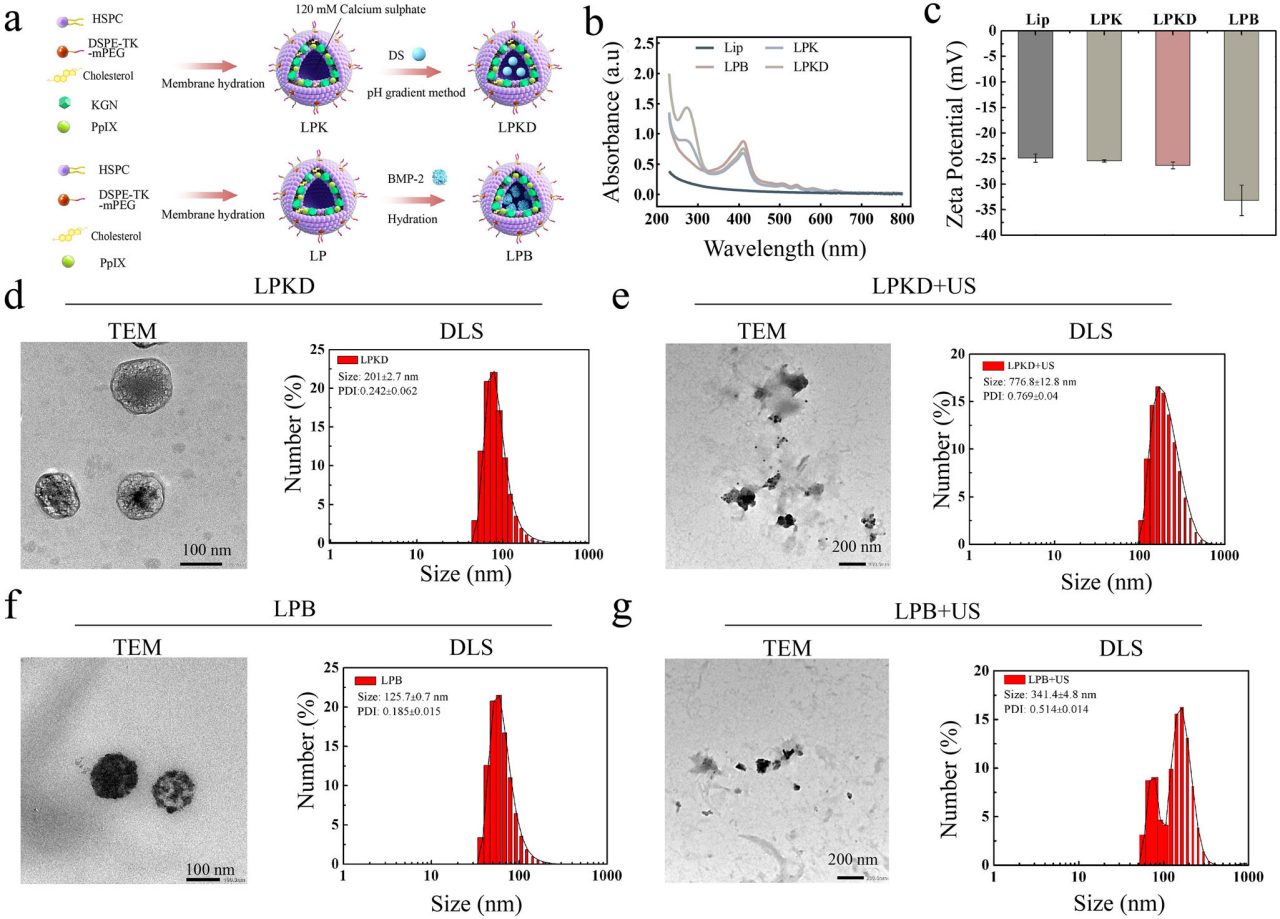
Synthesis and physicochemical properties of ROS‐responsive nanoliposomes with multi‐factors loading. a) Schematic diagram of the LPKD and LPB nanoparticle preparation process. b) UV spectra of Lip, LPK, LPKD and LPB nanoparticles at 200–800 nm. c) Zeta potential of Lip, LPK, LPKD and LPB nanoparticles. d,e) Representative TEM and size distribution of the LPKD nanoparticles with or without ultrasound treatment. f,g) Representative TEM and size distribution of the LPB nanoparticles with or without ultrasound treatment.

Intelligent responsive liposome nanoparticles (NPs) have been widely used for spatiotemporal delivery and multi‐stage synergistic therapy of tissue regeneration.^[^ ^25^ ^]^ Herein, ROS‐responsive LPKD/LPB liposome NPs loaded with anti‐inflammatory drug DS / chondrogenic factor KGN (LPKD) and osteogenic protein BMP‐2 (LPB) were synthesized by filming‐rehydration and pH gradient methods as previously described^[^ ^23^ ^]^ (**Figure** **1**a), respectively. To confirm the effective loading of LPKD/LPB nanoparticles, qualitative analysis was performed using UV spectrum. The results (Figure 1b) showed an absorption peak of KGN at 278 nm for LPK and an absorption peak of KGN and DS at wavelengths of 278 and 270 nm. Moreover, the encapsulation rate of DS and KGN for LPKD nanoparticles was calculated 92.2% and 45.3%, respectively, indicating successful drug loading. In addition, the loading rate of BMP‐2 for LPB nanoparticles was measured to 32.6% using an ELISA kit because that there is no special absorption peak for LPB (Figure S1a, Supporting Information). Zeta potential values were also measured, revealing −24.91 ± 0.781 mV for Lip, −25.01 ± 0.22 for LPK, −26.33 ± 0.65 mV for LPKD and −33.16 ± 3.01 mV for LPB (Figure 1c). Notably, the zeta potential value of LPB was lower than those of Lip, LKP and LPKD groups, demonstrating that the negatively charged BMP‐2 protein could decrease the charge of composite nanoliposomes. As shown in Figure 1d,f, TEM images revealed that LPB/LPKD nanoparticles exhibited a uniform spherical structure with sizes of 116.3 ± 0.8 nm and 142.6 ± 1.3 nm, respectively. While DLS measurements indicated that the mean diameters of LPB/LPKD nanoparticles were 125.7 ± 0.7 nm (PDI = 0.185 ± 0.015) and 201 ± 2.7 nm (PDI = 0.242 ± 0.062), respectively. The hydrodynamic size observed by DLS was slightly larger than the dry size measured by TEM, which might be due to the fact that DLS detects the hydrodynamic size, including the hydration layer and surface coating of the liposomes, while TEM provides a measurement of the surface profile of the liposomes. To assess the stability of LPB/LPKD nanoparticles, the mean diameters measured using DLS after being sitting for 7 days (Figure S1b, Supporting Information). The results demonstrated LPB/LPKD nanoparticles had a high structural stability, as indicated by no significant changes of the DLS size. More importantly, in order to prove the ROS‐responsive rupture of LPB/LPKD nanoparticles, TEM images and DLS measurement of LPB/LPKD nanoparticles after ultrasound (US) treatment for 3 min were conducted. The results (Figure 1e,g) displayed that the breakdown of the spherical structure and the aggregation of NPs with DLS size increasing to 341.4 ± 4.8 nm (PDI = 0.514 ± 0.014) and 776.8 ± 12.8 nm (PDI = 0.769 ± 0.04) after US treatment, which indicated that ultrasonic stimulation triggered ROS generation, further resulting in TK bonding cleavage and rupture of LPB/LPKD nanoparticles.

### 2.2 Preparation and Characterization of In Situ Nanocomposite Hydrogel Doped with ROS‐Responsive Nanoliposome

**Figure 2:**
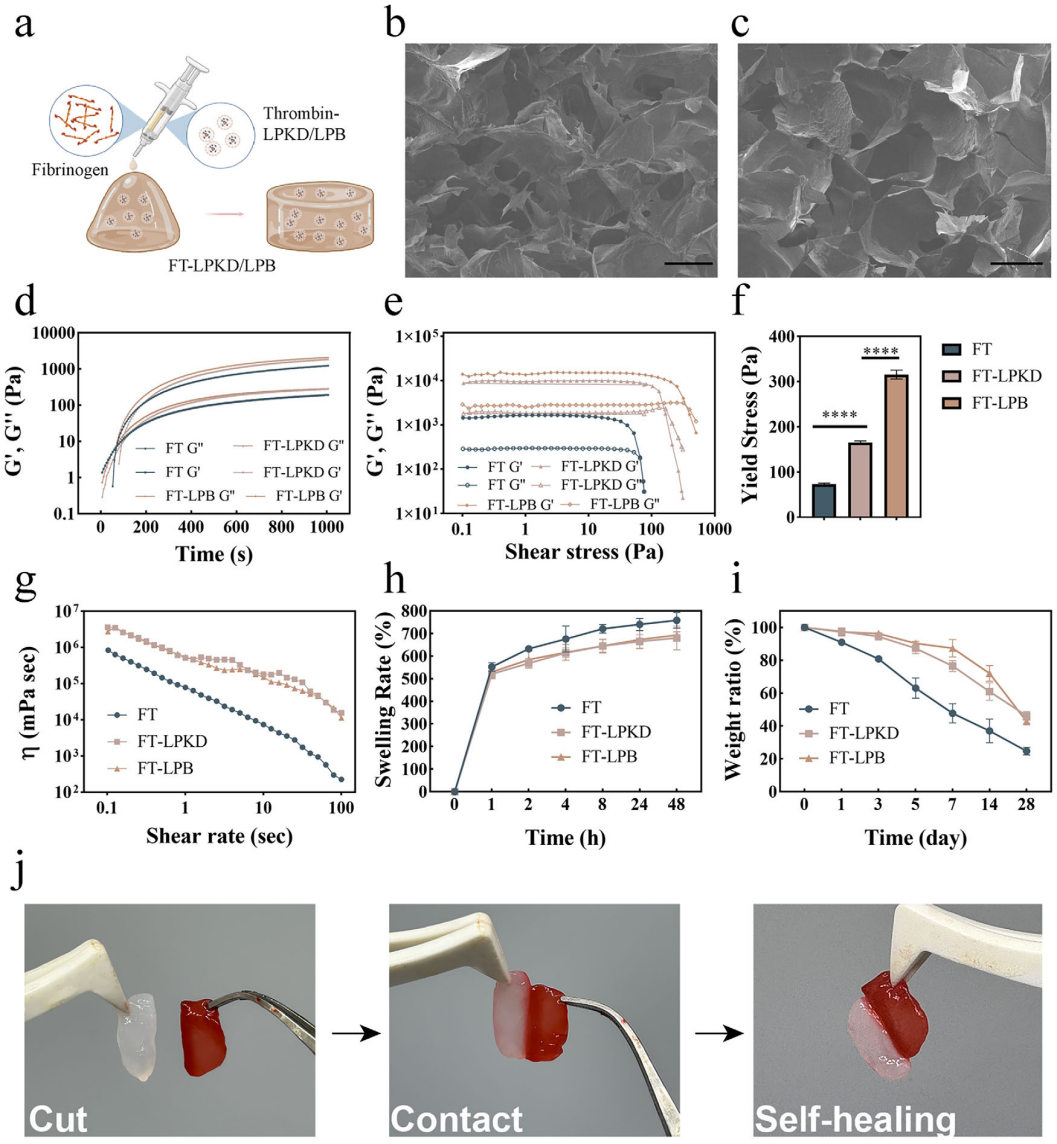
Characterization of in situ nanocomposite hydrogel. a) Schematic diagram of the preparation of FT‐FLPKD/LPB in situ hydrogel. b) Photographs of the FT‐FLPKD hydrogel before and after gelation. c) SEM images of FT‐LPB and FT‐LPKD nanocomposite hydrogels (Scale bar: 50 µm). d) Viscoelasticity and gelation kinetics. e,f) Elasticity modulus and yied stress. g) Shear thinning. h) Swelling rate of the FT, FT‐LPB and FT‐LPKD nanocomposite hydrogels. i) Degradation properties of the FT, FT‐LPB and FT‐LPKD hydrogels. j) Self‐healing behavior of FT‐LPKD nanocomposite hydrogels. (Data are presented as mean ± SD. n = 3. *p < 0.05, **p < 0.01, ***p < 0.001 and ****p < 0.0001).

In situ nanocomposite FT‐LPKD/LPB hydrogels were fabricated with the enzymatic reaction of fibrinogen and thromin.^[^ ^26^ ^]^ In which, pre‐synthetic LPKD and LPB naoliposomes were doped into the hydrogel precursor to construct a ROS‐responsive drug delivery platform (**Figure** **2**a). As shown in Figure 2b, in situ gelatin of FT‐LPKD nanocomposite hydrogels occurred within 2 min. SEM images (Figure 2c) showed that FT‐LPKD/LPB hydrogels had interconnected and evenly distributed porous structure, which are beneficial for cell adhesion and migration. Moreover, the corresponding EDX mapping (Figure S2, Supporting Information) exhibited the signal of P, Cl, S elements enhanced due to the addition of the LPKD liposomes, which indicated liposomes were evenly distributed within the FT‐LPKD/LPB hydrogels. Additionally, the rheological behavior of the FT‐LPB/LPKD hydrogels was evaluated to determine the effect of LPB/LPKD nanoparticles on the gelation process. Figure 2d showed that the storage modulus (G′) began to exceed the loss modulus (G″) at the time of 70–75 s upon two precursor aqueous solution of fibrinogen and thrombin‐LPB/LPKD connected, indicating the initiation of the gelation process from liquid‐to‐gel. After gelation, the storage moduli (G′) of all hydrogels groups were significantly higher that the corresponding loss modulus (G″) within a certain range (10^−1^–10^2^ Pa) of shear stress (Figure 2e), which revealed all prepared hydrogels had elastic property. Moreover, the G″ values in FT‐LPB/LPKD hydrogels were significantly higher than that of FT hydrogels, suggesting that FT‐LPB/LPKD hydrogels had more superior viscoelasticity. With the increase of shear stress, the G″ and G′ moduli remains nearly constant in parallel until reaching a specific stress threshold (i.e., yield stress). As shown in Figure 2f, the yield stress of FT‐LPB/LPKD hydrogels was significantly higher than that of FT hydrogels, indicating the doping of LPB/BPKD nanoliposomes significantly enhanced the mechanical stability of FT‐LPB/LPKD hydrogels. Moreover, the shear viscosity (η, Figure 2g) of all three FT/ FT‐LPB/ FT‐LPKD hydrogels gradually decreased with the increase of shear rate, which revealed all these three kinds of hydrogels had shear thinning property. The swelling rate (Figure 2h) of FT‐LPB/LPKD hydrogels was determined with weighing method. The results showed that the swelling rate reached to 664.95% and 672.67%, respectively, at swelling equilibrium in DPBS at 37 °C after 24 h. Moreover, proper biodegradation of hydrogels plays an important role in drug delivery and tissue regeneration. To evaluate their biodegradation properties, the hydrogels were incubated in DPBS at 37 °C for 28 days. At every time point, the remaining weights of the FT and FT‐LPB/LPKD hydrogels were determined after lyophilization. The results showed that FT‐LPB/LPKD hydrogels retained 42.76% and 46.13% of their original mass after 28 days in DPBS, respectively, while the FT hydrogel mass ratio only retained 24.72% (Figure 2i). The results indicated that the degradation rate of FT‐LPB/LPKD nanocomposite hydrogel was lower than that of fibrin hydrogel, likely due to hydrogen bonding formed between NH_2_‐ and OH‐ groups in the fibrin and DSPE‐TK‐mPEG/HSPC mixture.^[^ ^27^ ^]^ The formed hydrogen bonding could enhance intermolecular forces, further promoted the mechanical strength and reduced the degradation rate of nanocomposite FLPT/FLPKT hydrogels.^[^ ^28^ ^]^ More importantly, we also confirmed the self‐healing property of FT‐ LPKD hydrogels using a cut and heal test. As shown in Figure 2j, this type of hydrogels was able to self‐heal after being cut and re‐connected.

### 2.3 Fabrication and Characterization of Multi‐Factors Loaded Nanohydrogels Composite Bilayer Scaffolds with ROS‐Controlled Release

**Figure 3:**
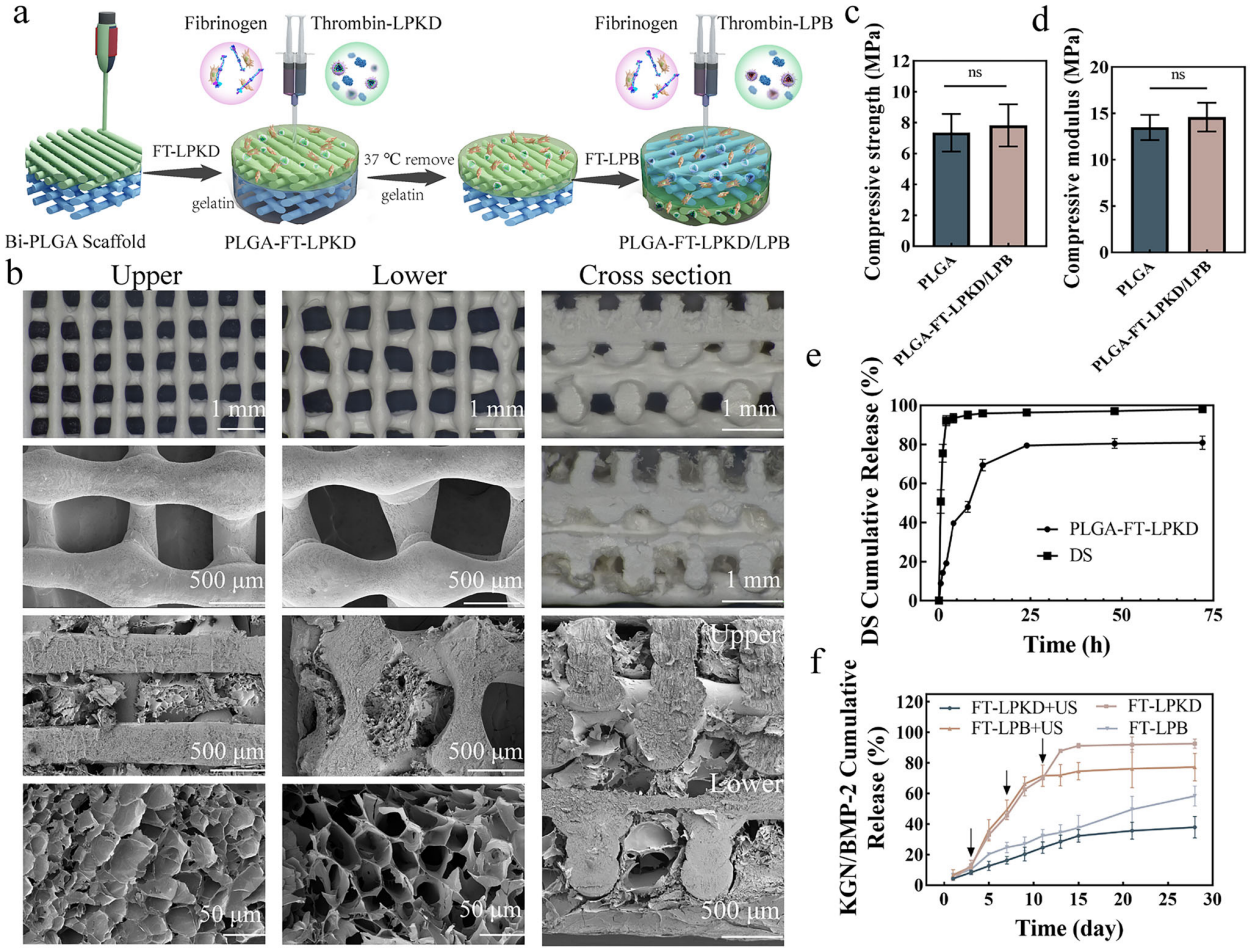
Fabrication and performance of multi‐factors loaded FT‐LPKD/LPB nanohydrogel composite bi‐layer PLGA scaffolds. a) Schematic diagram of the fabrication of PLGA‐FT‐LPKD/LPB nanohydrogel composite scaffolds. b) SEM images of PLGA‐FT‐LPKD/LPB nanohydrogel composite scaffolds on the upper, lower and cross section. c,d) The compressive strength and compressive modulus of PLGA bilayer scaffold and PLGA‐FT‐LPKD/LPB nanohydrogel composite bilayer scaffolds. e) The cumulative release of DS from PLGA‐FT‐LPKD nanohydrogel scaffolds and DS solution at 0.5, 2, 4, 8, 12, 24, 48, and 72 h. f) Cumulative release of KGN and BMP‐2 at the day of 1, 3, 5, 7, 9, 11, 13, 15, 21, 28 with or without ultrasound treatment. (Data are presented as mean ± SD. n = 3. *p < 0.05, **p < 0.01, ***p < 0.001 and ****p < 0.0001).

The PLGA bilayer scaffolds with two pore size to mimic the natural osteochondral structure were 3D printed by cryogenic deposition.^[^ ^29^ ^]^ After that, the precursors including thrombin‐LPKD/LPB and fibrinogen were injected into the upper layer pore (PLGA‐FT‐LPKD) and lower layer pore (PLGA‐FT‐LPB) of bilayer PLGA scaffold to prepare multi‐factors loaded nanohydrogels composite bilayer scaffolds via enzymatic reaction using gelatin placeholder method (**Figure** **3**a). SEM images displayed their microstructure of the nanohydrogels composite bilayer scaffolds from different angles. As shown in Figure 3b, the complex hydrogel scaffolds consisted of upper and lower layer with regular interconnected porous structure, providing spatial mechanical support and 3D cell culture microenvironment for tissue growth. The upper layer composed of PLGA hard scaffolds with a filament spacing of 300 µm and FT‐LPKD hybrid soft hydrogels, enabling the cascade release of small molecule water‐soluble DS to suppress early inflammation during OA development. Moreover, the FT‐LPKD hybrid hydrogel serves as a ROS‐controlled drug delivery system for KGN, promoting cartilage regeneration through external ultrasound stimulation. The lower layer is composed of PLGA hard scaffold with a filament spacing of 500 µm and FT‐LPB hybrid hydrogel, which promotes the controlled release of BMP‐2 protein, promoting subchondral bone regeneration. Additionally, the mechanical properties (Figure 3c,d) of the PLGA‐FT‐LPKD/LPB scaffolds were assessed. The mean compressive strength and compression modulus of PLGA‐FT‐LPKD/LPB nanohydrogel composite scaffolds were 8.3 and 15.3 MPa, respectively. While PLGA bi‐layer scaffolds exhibited slightly lower compressive strength and compression modulus with values of 7.2 and 13.2 MPa. These results indicated the coating of FT‐LPKD/LPB hydrogel contributed slightly on the mechanical properties of PLGA scaffolds, but will make a huge contribution on the 3D microenvironment culture and dual‐lineage differentiation of MSCs in future.

### 2.4 Sequential Release of Multi‐Factors Loaded in Nanohydrogels Composite Bilayer Scaffolds

Spatiotemporal sequential drug delivery strategies play a vital role in the multi‐stage synergistic therapy of osteochondral defects.^[^ ^30^ ^]^ To trigger stepwise responses, including anti‐inflammation, chondrogenesis and osteogeneis, in the multi‐stage osteochondral regeneration process, anti‐inflammatory drug (DS) was effectively loaded into LPKD nanoparticles by pH gradient method, enabling rapidly release to inhibit early‐stage inflammatory reaction. Chondrogenic factor (KGN) and osteogenic protein (BMP‐2) were loaded into LPKD/LPB nanoparticles via filming‐rehydration method, which could achieve spatiotemporal controlled release via ultrasound stimulation to impove dual‐lineage differentiation of MSCs for osteochondral regeneration. As shown in Figure 3e, the PLGA‐FT‐LPKD nanohydrogel composite scaffold displayed a burst release of water‐soluble DS with the cumulative release of DS reaching 79.48% within 24 h, while the pure DS solution as a positive control showed quick release of DS reaching 95.85% within 12 h. After 48 h, the cumulative release curves of DS from the PLGA‐FT‐LPKD nanohydrogel composite scaffold gradually reach a plateau of 80.87%. The rapidly release of DS from the nanohydrogel composite scaffolds provides an excellent delivery strategy in the inflammatory phase of osteochondral defects without any extra stimulation. Additionally, the encapsulation efficiencies of KGN and BMP‐2 loaded into LPKD and LPB were determined using HPLC and an ELISA kit, respectively, resulting in encapsulation efficiencies of 86.23% and 36.16%. Moreover, the spatiotemporal sequential release of KGN and BMP‐2 from PLGA‐FT‐LPK and PLGA‐FT‐LPB, respectively, was controlled by ultrasound treatment (1 W cm^−2^, 3 min). Figure 3f showed that the release profiles of KGN and BMP‐2 from PLGA‐FT‐LPK and PLGA‐FT‐LPB nanohydrogel composite scaffolds over a 28‐day period, respectively. A small release of the fat‐soluble KGN with ≈8.33% was detected for the first three days. Following ultrasound stimulation at day of 3, 7 and 11, the release of KGN significantly increased to reach a cumulative release of 91.30% by day of 15. In contrast, the PLGA‐FT‐LPKD nanohydrogel composite scaffold without ultrasound stimulation exhibited a slower sustained release of KGN with a cumulative release of 38.74% at 28 days. Similarly, the release kinetics of BMP‐2 from the PLGA‐FT‐LPB nanohydrogel composite scaffold followed a similar trend with a cumulative release of 72.18% by the day of 13 under ultrasound stimulation.

### 2.5 3D Culture, Migration and Biocompatibility Evaluation within Nanohydrogels Composite Bilayer Scaffolds

**Figure 4:**
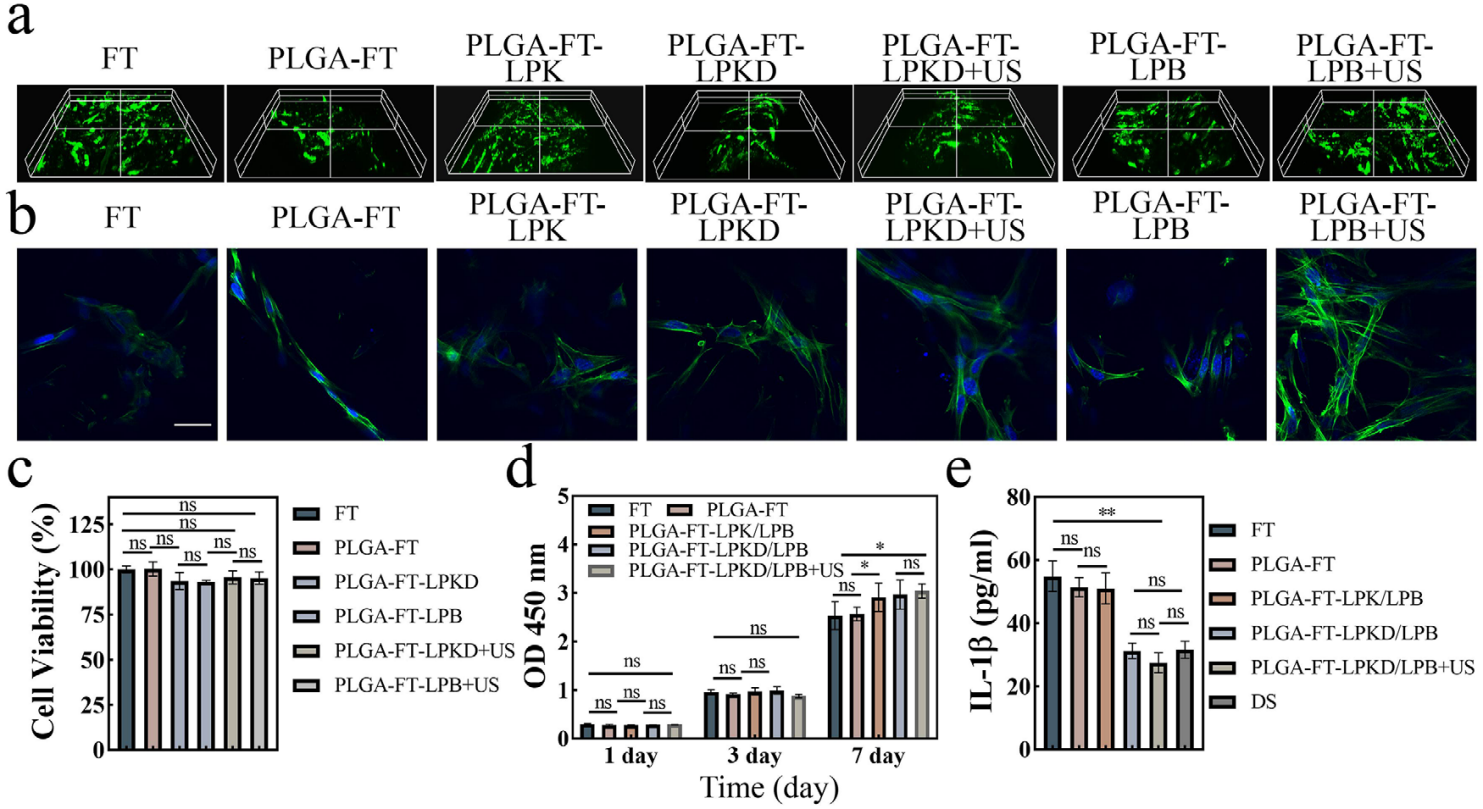
3D culture of BMSCs and biocompatibility of multi‐factors loaded nanohydrogels composite bilayer scaffolds. a) Live/dead staining images within nanohydrogel composite bilayer scaffolds after being cultured for 3 days. b) Cytoskeletal morphologies of BMSCs in hydrogel composite 3D scaffolds (Scar bar: 50 µm). c) Cytotoxicity detection of multi‐factors loaded nanohydrogel composite bilayer scaffolds. d) Cell proliferation of BMSCs in nanohydrogel composite 3D scaffolds for 1, 3 and 7 days. e) The expression of anti‐inflammatory factor IL‐1*β* in nanohydrogel composite 3D scaffolds by ELISA Kit. (Data are presented as mean ± SD. n = 3. *p < 0.05, **p < 0.01, ***p < 0.001 and ****p < 0.0001).

To evaluate 3D culture of BMSCs within PLGA‐FT‐LPKD/LPB nanohydrogels composite bilayer scaffolds, live/dead staining images and FITC/DAPI staining cytoskeletal morphologies were observed after being cultured for 3 days. As shown in **Figure** **4**a, almost all BMSCs remained viable and distributed well within 3D hydrogel‐like ECM microenvironment of the PLGA‐FT‐LPKD/LPB nanohydrogels composite bilayer scaffolds. Furthermore, the cytoskeletal morphologies (Figure 4b) of BMSCs in the different groups displayed BMSCs spread well with a number of cellular pseudopods in the PLGA‐FT‐LPKD/LPB nanohydrogels composite bilayer scaffolds.

Additionally, it is essential to detect BMSCs migration around the PLGA‐FT‐LPKD/LPB nanohydrogels composite bilayer scaffolds due to the dual‐lineage differentiation ability of BMSCs to chondrocytes and osteoblasts.^[^ ^31^ ^]^ The results (Figure S3a, Supporting Information) showed that BMSC migration rate in the PLGA‐FT‐LPKD/LPB+US group was significantly higher than that in the control groups after co‐culture with serum‐free medium for 12 and 24 h. The residual area (remaining area/total area × 100%) was calculated by ImageJ software. As shown in Figure S3b (Supporting Information), the residual area gradually decreased over time. The residual area of BMSCs in the PLGA‐FT‐LPKD/LPB+US group (1.32 ± 0.085 mm^2^) and the PLGA‐FT‐LPKD/LPB group (1.61 ± 0.032 mm^2^) was significantly smaller than that in the FT group (2.464 ± 0.094 mm^2^) after 24 h of co‐culture, indicating migration rate of BMSCs gradually increased.

Furthermore, the viability of BMSC in PLGA‐FT‐LPKD/LPB+US nanohydrogel composite scaffolds was detected by the CCK‐8 kit (Figure 4c). The results indicated BMSCs had high cell viability ranging from 93.04% to 95.61% and no significant toxicity in different groups with or without ultrasound stimulation, suggesting PLGA‐FT‐LPKD/LPB nanohydrogel composite scaffolds had excellent biocompatibility. The proliferation of BMSCs in PLGA‐FT‐LPKD/LPB nanohydrogel composite scaffolds was also assessed via the CCK‐8 at day of 1, 3 and 7. As shown in Figure 4d, BMSCs exhibited better proliferation capability within nanohydrogel composite scaffolds than that of FT group, which might be attributed to the successful construction of soft and hard matrix complexes, enhancing cell proliferation by mimicking ECM microenvironment.^[^ ^32^ ^]^

### 2.6 Anti‐Inflammatory Performance of Multi‐Factors Loaded in the Nanohydrogels Composite Bilayer Scaffolds In Vitro

**Figure 5:**
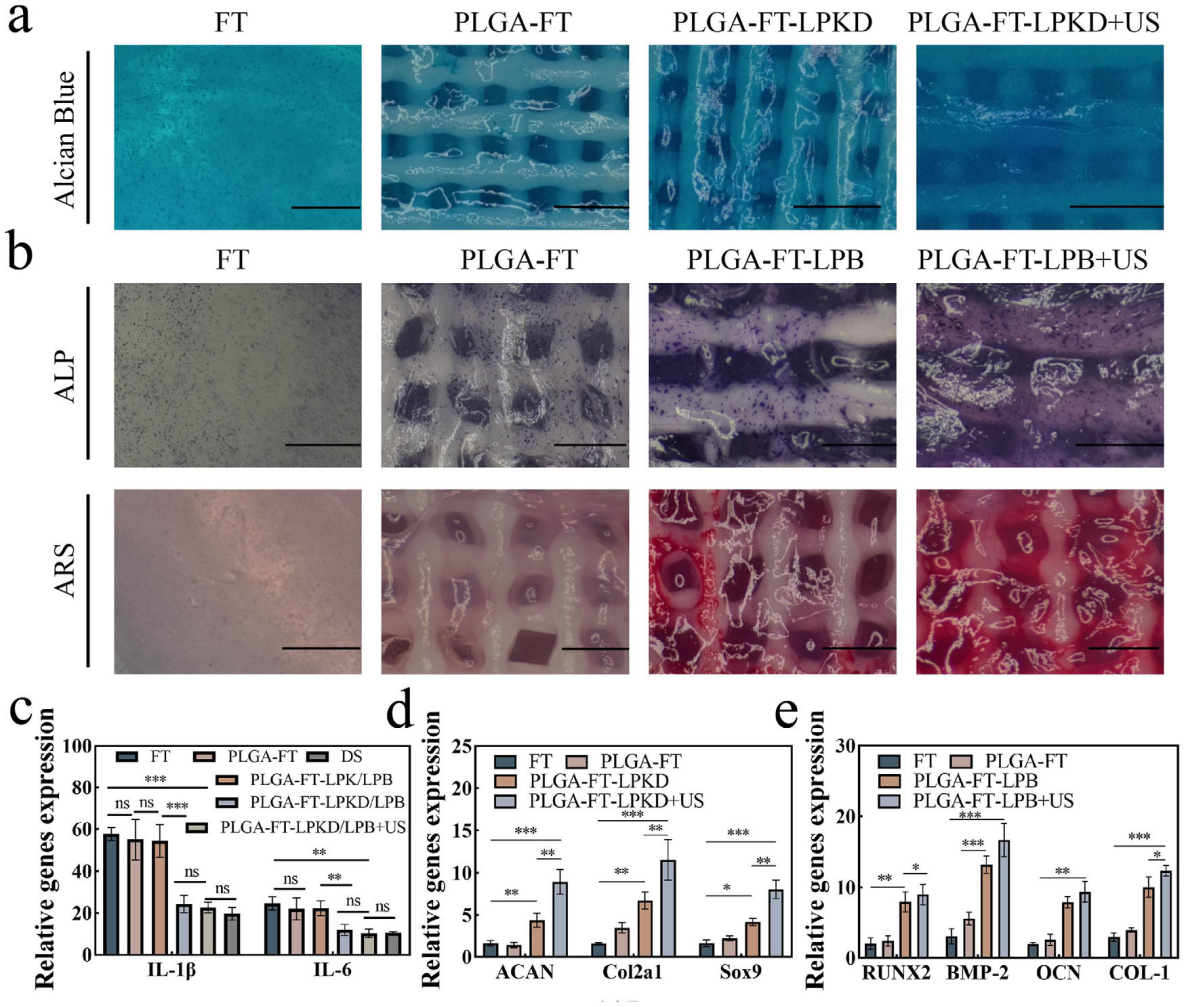
Evaluation of osteogenic and chondrogenic dual‐linage differentiation in vitro with multifactor‐loaded nanocomposite hydrogel composite bilayer scaffolds. a) Alcian blue staining were used to evaluate chondrogenic differentiation of BMSCs within the upper layer of PLGA‐FT‐LPKD hydrogel composite scaffolds at 7 days (Scale bar: 1000 µm). b) ALP and ARS staining were used to evaluate osteogenic differentiation of BMSCs within the lower layer of PLGA‐FT‐LPB hydrogel composite scaffolds at 14 days (Scale bar: 1000 µm). c) Anti‐inflammatory gene expression of IL‐1*β* and IL‐6 at the day of 3. d) Chondrogenic gene expression levels of ACAN, Col2al and Sox9 at the day of 14. e) Osteogenesis gene expression of RUNX2, BMP‐2, OCN and Col‐1 at the day of 14. (Data are presented as mean ± SD. n = 3. *p < 0.05, **p < 0.01, ***p < 0.001 and ****p < 0.0001).

To evaluate the in vitro anti‐inflammatory effect of 3D‐bioprinted PLGA bilayer composite scaffolds coated with FT‐LPK and FT‐LPKD, BMSCs were cultured in TNF‐α induced inflammatory medium for 3 days and measured by ELISA Kit. The results (Figure 4e) indicated that the FT‐LPKD nanohydrogel composite scaffold significantly reduced IL‐1*β* level compared to that of FT group. Furthermore, no statistically significant difference in IL‐1*β* levels was observed after ultrasound treatment (1 W cm^−2^, 3 min), indicating that the PLGA‐FT‐LPKD/LPB scaffolds under ultrasound stimulation do not promote an inflammatory response. Interestingly, IL‐1*β* expression of BMSCs both in PLGA‐FT‐LPKD/LPB and PLGA‐FT‐LPKD/LPB+US nanohydrogel composite scaffolds was similar with that of DS group. In addition, the expression levels of the relative genes (IL‐1*β*, IL‐6) were detected via qRT‐PCR. The results (Figure 5c) showed that the gene expression of IL‐1*β* and IL‐6 was considerablely downregulated in both PLGA‐FT‐LPKD/LPB and PLGA‐FT‐LPKD/LPB+US nanohydrogel composite scaffolds. No statistically significant differences were observed between PLGA‐FT‐LPKD/LPB, PLGA‐FT‐LPKD/LPB+US nanohydrogel composite scaffolds and DS group.

### 2.7 In Vitro Dual‐Linage Differentiation of BMSCs in Nanohydrogels Composite Bilayer Scaffolds with ROS‐Controlled Sequential Release

The bi‐layer structure and sequential release of multi‐factors from PLGA‐FT‐LPKD/LPB nanohydrogel composite scaffolds play a vital role in the dual‐lineage differentiation of stem cells for osteochondral regeneration. Specifically, the upper PLGA‐FT‐LPKD complex hydrogel scaffolds aim to induce cartilage regeneration, while the lower PLGA‐FT‐LPB complex scaffolds seek to improve bone regeneration. Hence, we evaluated the chondrogenic and osteogenic differentiation effect of BMSCs within the upper and lower layer of PLGA‐FT‐LPKD/LPB nanohydrogel composite scaffolds, respectively. For chondrogenic differentiation ability of BMSCs in the upper PLGA‐FT‐LPKD hydrogel scaffold, Alcian blue staining images (**Figure** **5**a) displayed the highest expression of glycosaminoglycans in BMSCs cultured in the upper PLGA‐FT‐LPKD nanohydrogel composite scaffolds for 7 days, indicating strong chondrogenic differentiation potential. Moreover, the expression levels of the typical chondrogenic gene markers including ACAN, Sox9 and Col‐II were detected with qRT‐PCR via culturing BMSCs within the PLGA‐FT‐LPKD scaffold for 14 days. The results (Figure 5d) showed that the expression levels of ACAN, Sox9 and Col‐II increased 2.03‐, 1.92‐ and 1.72‐fold, respectively, compared to the non‐ultrasound group. Furthermore, we also evaluated the chondrogenic differentiation level of PLGA‐FT‐LPK groups without the doping of DS. Alcian blue staining images (Figure S4a, Supporting Information) showed that PLGA‐FT‐LPK group after US treatment had a higher expression. The qRT‐PCR results (Figure S4b, Supporting Information) also indicated that chondrogenic gene expression levels of ACAN, Sox9 and Col‐II within PLGA‐FT‐LPK + US group were higher than that of FT group, suggesting its excellent cartilage regeneration capacity. Moreover, the ACAN, Sox9 and Col‐II genes expression levels of PLGA‐FT‐LPK + US decreased to 87.73%, 87.09% and 84.68% as compared to the PLGA‐FT‐LPKD + US group, suggesting that hydrogel composite scaffolds can improve the expression of chondrogenic genes and promote cartilage regeneration after inhibiting inflammation.

Besides, osteogenic differentiation capacity of BMSCs within the PLGA‐FT‐LPB hydrogel composite scaffolds was evaluated by performing ALP and Alizarin red staining after being cultured for 7 and 14 days, respectively. ALP staining images (Figure 5b) demonstrated stronger staining intensity within the PLGA‐FT‐LPB + US hydrogel composite scaffold than that of PLGA‐FT‐LPB group. Moreover, the quantification analysis by ALP activity kit (Figure S5a, Supporting Information) indicated that the ALP activity was considerably increased in the PLGA‐FT‐LPB + US group as compared with the PLGA‐FT‐LPB group, suggesting the PLGA‐FT‐LPB hydrogel complex scaffold can enhance differentiation of BMSCs to osteoblasts with ultrasound treatment. Furthermore, ARS staining images (Figure 5b) and corresponding quantitative analysis (Figure S5b, Supporting Information) also showed denser mineral nodules and a higher mineralization rate within the PLGA‐FT‐LPB + US group as compared to those of the PLGA‐FT‐LPB and control groups, indicating BMSCs within the PLGA‐FT‐LPB + US group had excellent late osteogenic differentiation ability. Besides, the effect of the PLGA‐FT‐LPB hydrogel composite scaffolds on osteogenic differentiation of BMSCs was analyzed by measuring the expression levels of typical osteogenic gene markers including ALP, BMP‐2, OCN and Col I via qRT‐PCR. The results (Figure 5e) showed that the expression levels of ALP, BMP‐2, OCN and Col I were significantly higher within the PLGA‐FT‐LPB + US group compared to non‐ultrasound treatment group, with respective changes of 1.37‐, 1.22‐, 1.13‐ and 1.27‐ folds. It is indicated that the PLGA‐FT‐LPB hydrogel composite scaffolds could significantly enhance osteogenic differentiation of BMSCs under ultrasonic stimulation, which is consistent with ALP and ARS staining analysis. Thus, these results revealed that PLGA‐FT‐LPB hydrogel hydrogel complex scaffolds with US treatment have an excellent ability to induce osteogenic differentiation of BMSCs, facilitating subchondral bone regeneration after in vivo implantation.

### 2.8 Osteochondral Repair Efficacy of Multi‐Factors Loaded Nanohydrogel Composite Bilayer Scaffolds on Osteochondral Defect Model In Vivo

**Figure 6:**
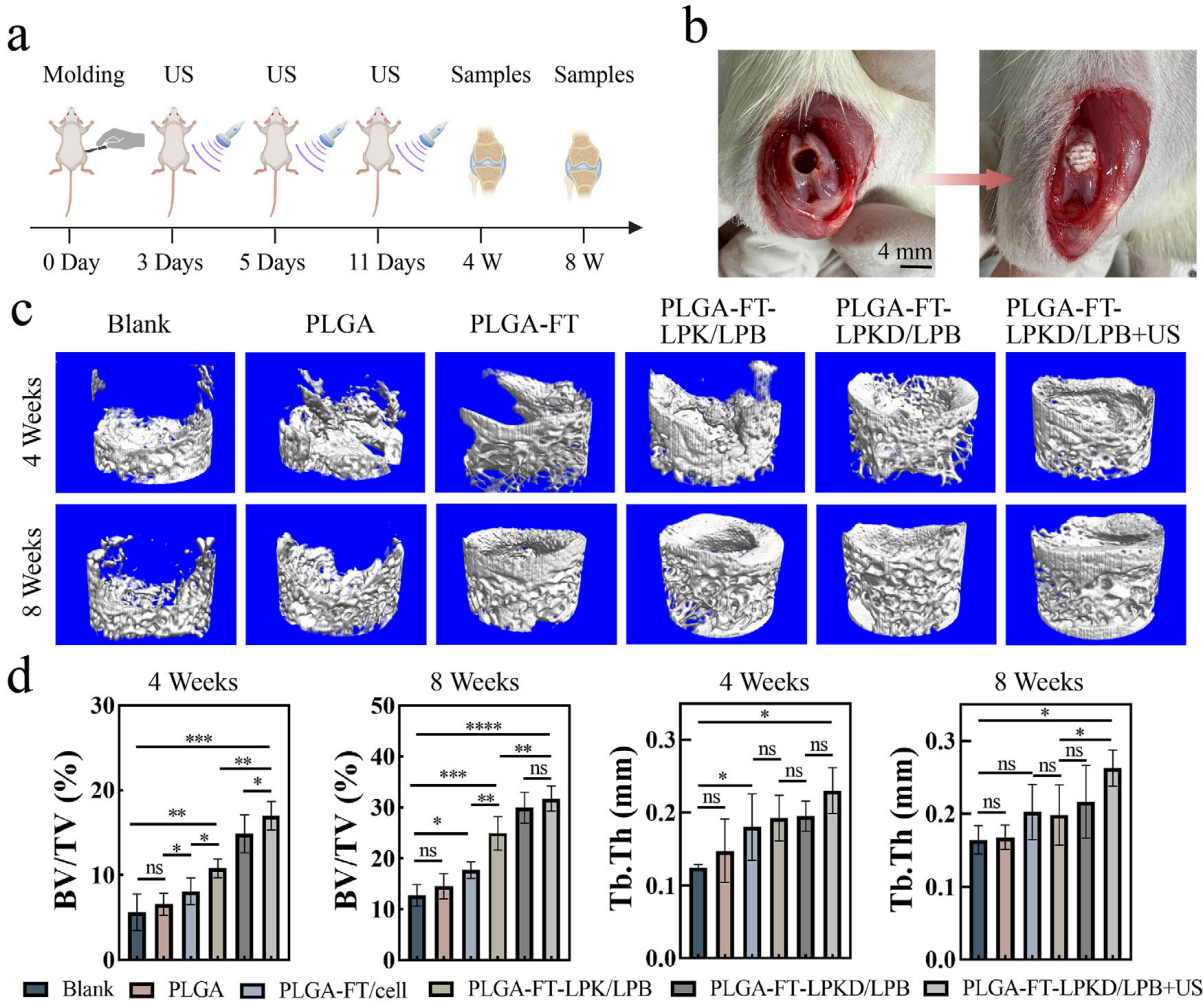
Effect of multifactor‐loaded nanocomposite hydrogel composite bilayer scaffolds on osteochondral repair in SD rats. a) The treatment schedule for the osteochondral defect model of SD rats in vivo. b) Images of PLGA‐FT‐LPKD/LPB scaffolds implanted in the osteochondral defect area. c) Micro‐CT images of bone defects in hydrogel composite scaffolds after implantation for 4 and 8 W. d) The quantitative data of micro‐CT confirmed by BV/TV and Tb. Th. (Data are presented as mean ± SD. n = 5. *p < 0.05, **p < 0.01, ***p < 0.001 and ****p < 0.0001).

To evaluate the capacity of PLGA‐FT‐LPKD/LPB+US hydrogel composite scaffolds to promote osteochondral regeneration in vivo, an osteochondral defect model with a diameter of 3.2 mm and a height of 3 mm was constructed in Sprague‐Dawley (SD) rats. The treatment schedule (**Figure** **6**a) for osteochondral defect therapy in SD rats is illustrated in this work. As shown in Figure 6b, these hydrogel composite scaffolds of PLGA, PLGA+FT, PLGA‐FT‐LPK/LPB, PLGA‐FT‐LPKD/LPB and PLGA‐FT‐LPKD/LPB+US were implanted into osteochondral defect site, respectively. All the rats were sacrificed after 4 and 8 W of implantation to harvest the joint samples. Moreover, regeneration tissue and tightly bound with the surrounding cartilage were observed around the defect area of PLGA‐FT‐LPK/LPB+US and PLGA‐FT‐LPKD/LPB+US groups. As shown in Figure S6 (Supporting Information), the PLGA, PLGA+FT, PLGA‐FT‐LPK/LPB groups were mostly filled with new tissues as well. However, the blank group exhibited an evident cavity due to limited self‐repair capacity. On the contrary, PLGA‐FT‐LPKD/LPB+US group exhibited a tight, cartilage‐like surface without collapse at the defect site after 8 W of implantation, indicating the PLGA‐FT‐LPKD/LPB hydrogel composite scaffold with US treatment had an excellent regeneration effect compared to other groups.

The micro‐CT images (Figure 6c) and corresponding quantitative data (Figure 6d) were used to assess the ability subchondral bone regeneration after 4 and 8 W of treatment. Micro‐CT images showed more new bone formation in the PLGA‐FT‐LPKD/LPB+US hydrogel composite scaffold compared to other groups. Moreover, the bone volume fraction (BV/TV) values showed an increasing trend in the PLGA‐FT‐LPKD/LPB+US group compared to other groups. Specifically, the BV/TV value was significantly higher in the PLGA‐FT‐LPKD/LPB+US scaffold group (16.92%) than these of the blank group (5.6%), the PLGA‐FT‐LPK/LPB+US group (14.81%) and the PLGA‐FT‐LPK/LPB group (10.8%) at 4 W. Moreover, the BV/TV value was further increased in the PLGA‐FT‐LPKD/LPB+US group (31.76%) compared to these of the blank group (12.75%), the PLGA‐FT‐LPK/LPB+US group (29.97%) and the PLGA‐FT‐LPK/LPB group (24.96%) at 8 W. Additionally, the trabecular number (Tb.Th) of the PLGA‐FT‐LPKD/LPB+US group was significantly higher than that of other groups at 4 and 8 W.

**Figure 7:**
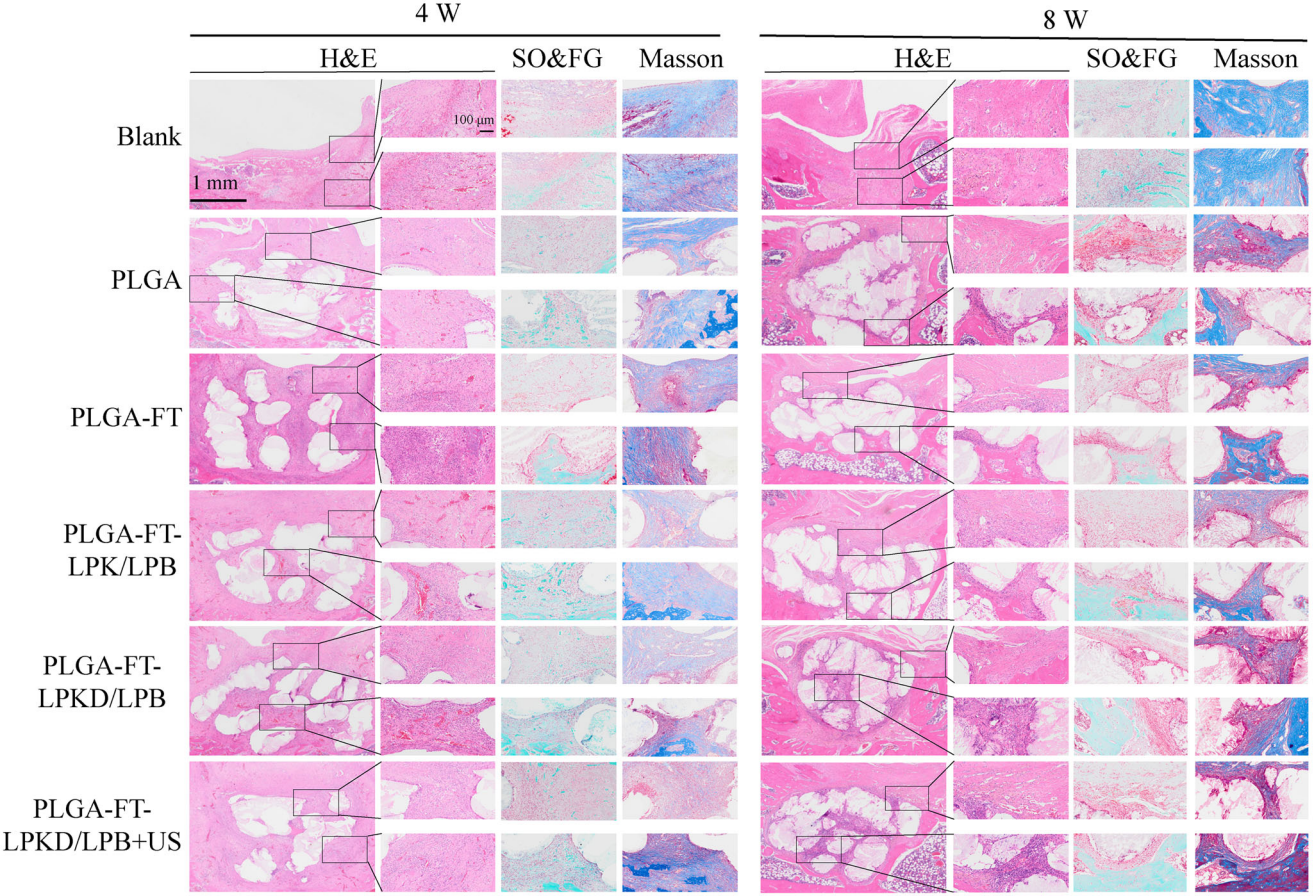
H&E, Safranin O/Fast Green and Masson staining images of SD rat joints at 4 and 8 W after treatment by ROS‐responsive hydrogel composite PLGA bilayer scaffolds.

For further investigating the regenerative efficacy of the hyaline cartilage and subchondral bone within PLGA‐FT‐LPKD/LPB complex scaffolds, histological analysis including Masson staining, safranine O/Fast green staining and H&E staining (**Figure** **7**) were carried out at 4 and 8 W after implantation. H&E staining images indicated that the PLGA groups was a large vacant space with few tissues at 4 W, whereas the defect region in PLGA‐FT‐LPKD/LPB and PLGA‐FT‐LPKD/LPB +US groups were covered with more new tissues compared to other groups. Moreover, much more new tissues were formed in the PLGA‐FT‐LPKD/LPB +US group at 8 W. In addition, safranine O/Fast green staining images displayed a significant presence of glycosaminoglycans and well‐integrated hyaline cartilage in the defect region of the FT‐LPKD/LPB +US group at 8 W. The regenerated cartilage showed that the typical hyaline cartilage morphology with band‐like chondrocyte distribution features, resembling the structure of normal cartilage. However, the hyaline cartilage in the blank and PLGA groups defect region was lacking and few glycosaminoglycans were found in the PLGA+FT, PLGA‐FT‐LPK/LPB groups. Besides, Masson staining images showed that a substantial amount of new bone tissues were formed around the PLGA‐FT‐LPKD/LPB +US hydrogel composite scaffold. In contrast, the blank and PLGA groups showed a thinner layer of new bone tissue at 8 W. These results of histological analysis demonstrated that PLGA‐FT‐LPKD/LPB +US hydrogel composite scaffolds distinctly facilitated the reconstruction of osteochondral tissue. More importantly, H&E staining images (Figure S7, Supporting Information) of the major organs (e.g., heart, liver, spleen, lung and kidney) after 8 W of implantation demonstrated the PLGA‐FT‐LPKD/LPB hydrogel composite scaffolds had an excellent long‐term biosafety.

**Figure 8:**
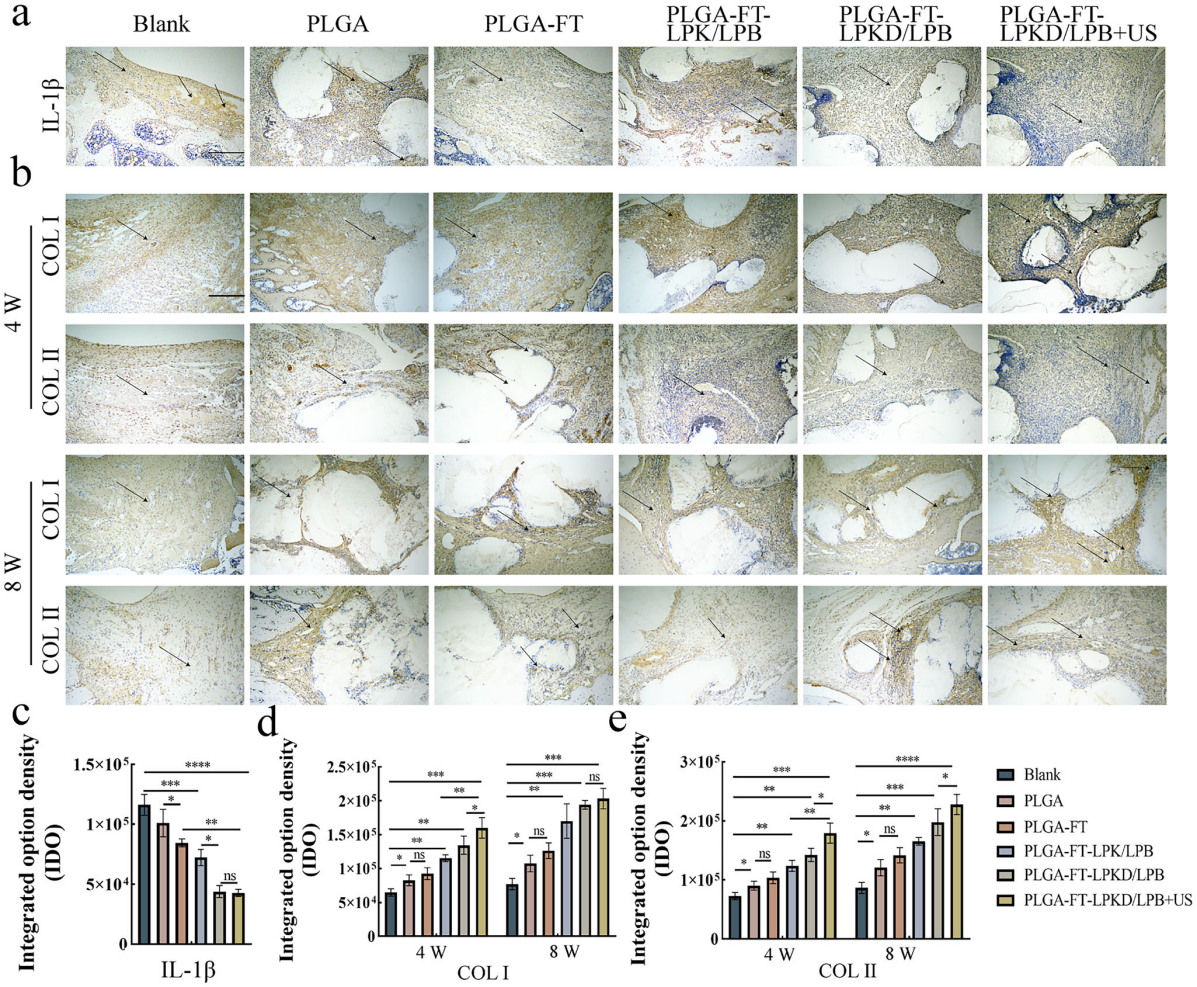
Immunohistochemical staining analysis of osteochondral defect after treatment by ROS‐responsive hydrogel composite PLGA bi‐layer scaffolds. a) Expression of the IL‐1*β* at 4 W (Scale bar: 200 µm). b) Expression of collagen I and collagen II at 4 and 8 W (Scale bar: 200 µm). c) The semi‐quantitative analysis of anti‐inflammatory factors IL‐1*β*. d,e) The semi‐quantitative analysis of collagen I and collagen II at 4 and 8 W. (Data are presented as mean ± SD. n = 5. *p < 0.05, **p < 0.01, ***p < 0.001 and ****p < 0.0001).

Additionally, the control group displayed significant higher expression of the inflammatory cytokine IL‐1*β* on the osteochondral defect surface, indicating severe inflammatory responses in the process of osteochondral defects. As shown in **Figure** **8**a, inflammatory cells and fibrous tissue were visible on the control group's osteochondral defect surface. Both the PLGA‐FT‐LPKD/LPB and PLGA‐FT‐LPKD/LPB+US scaffold treatment groups showed a significant decrease of IL‐1*β* expression level (Figure 8c) compared to that of the blank group, suggesting inflammatory management was DS‐dependent and had no additional effect by ultrasound treatment. Moreover, time‐course expression of cytokines (Figure S8, Supporting Information) further showed that the expression of pro‐inflammatory cytokines (IL‐1*β* and IL‐6) was highest on the 3rd days after surgery, and then the expression value of pro‐inflammatory cytokines was most rapidly decreased due to the release of DS from PLGA‐FT‐LPKD/LPB nanohydrogel scaffolds. Meanwhile, the expression amount of anti‐inflammatory cytokines (IL‐4 and IL‐10) was highest within the group of PLGA‐FT‐LPKD/LPB nanohydrogel scaffolds, especially on the 7th days after surgery, which could protect chondrocytes from inflammatory damage and promote cartilage extracellular matrix synthesis, thereby maintaining cartilage homeostasis.

Furthermore, COL I and COL II, as specific expression markers of bone and cartilage tissues, immunohistochemistry staining images (Figure 8b) of these two proteins were used to evaluated the capability of osteochondral regeneration. The results showed that both collagen I and collagen II were detected at 4 and 8 W, with COL II primarily distributed in the dense cartilage zone and COL I existed in the porous subchondral trabecular zone. Additionally, COL II was the predominant expression collagen in the new cartilage tissue in the upper layer of PLGA‐FT‐LPKD/LPB+US hydrogel composite bi‐layer scaffolds, indicating that hyaline‐like cartilage without fibrillation were newly formed. Most notably, a physiological osteochondral interface could be noticed in the PLGA, PLGA+FT, PLGA‐FT‐LPK/LPB, PLGA‐FT‐LPKD/LPB, PLGA‐FT‐LPKD/LPB+US groups as compared to blank group. Moreover, corresponding quantitative analysis (Figure 8d,e) showed the PLGA‐FT‐LPKD/LPB+US group had a higher expression of COL I and COL II, which could effectively induce the dual‐lineage regeneration of BMSCs in different zones, suggesting PLGA‐FT‐LPKD/LPB hydrogel composite scaffolds possess an excellent osteochondral regeneration capability.

The above analysis demonstrated that these ROS‐activated nanohydrogel scaffolds, with multi‐factors controlled release, could closely mimic soft‐hard complex matrix structure of osteochondral tissue to induce dual‐lineage regeneration of cartilage and subchondral bone tissue with long‐term biosafety in the osteochondral defect Model. Compared to traditional hydrogel scaffolds, these newly‐developed nanohydrogel scaffolds feature an enhanced gradient mechanical structure, surpassing that of pure hydrogels (such as GelMA), which could be widely applied in hierarchical structured soft‐hard tissue regeneration. More importantly, the nanohydrogel scaffolds offer a more closely 3D matrix microenvironments than traditional scaffolds (such as PCL), thereby supporting 3D cell cultivation instead of 2D culture on the wall surface of porous scaffolds. In brief, these newly‐developed nanohydrogel scaffolds not only provide spatial mechanical support through 3D‐printed complex structure but also recreate 3D matrix microenvironments with matrix‐mimic hydrogels. This combination shows promising applications and long‐term biosafety for the efficient regeneration of multi‐stage tissues. In addition, this nanohydrogel scaffolds are expected to be applied in the clinic in future due to their main ingredients, including fibrin derived from mammal and PLGA without any toxic to humans has been approved by FDA. However, the nanohydrogel scaffolds still face some limitations, such as the inability to undergo terminal moist heat Sterilization, complex preparation process restricting its industrial‐scale production.

## 3 Conclusion

In this study, we proposed a soft‐hard concept for preparing multi‐factors loaded FT‐LPKD/LPB nanohydrogel (as soft matrix) composite bilayer PLGA scaffolds (as hard matrix) by the combination of cryogenic deposition and gelatin placeholder method. As a result, these newly developed ROS‐activated nanohydrogel scaffolds achieved multi‐factors controlled release, including rapidly release of DS for 72 h and controlled release of KGN and BMP2 for 15 days under ultrasound stimulation to facilitate stepwise biological responses. More importantly, this novel kind of FT‐LPKD/LPB nanohydrogel composite bilayer scaffolds provided 3D culture and induce dual‐lineage differentiation of BMSCs in vitro, efficiently inhibited early‐stage inflammatory reaction for 4 weeks by rapidly releasing DS and improved excellent cartilage and subchondral bone tissue regeneration for 8 weeks in osteochondral defects model of SD rats in vivo. This study suggested a biomimetic innovation with such specific soft‐hard bilayer structure and sequential delivery of functional factors, offering a new solution for multi‐stage regeneration of complex tissues.

## 4 Experimental Section

### Materials

Kartogenin (KGN, 317.338 g mol^−1^), Protoporphyrin IX (PpIX, 562.66 g mol^−1^) and Human Bone Morphogenetic Protein‐2 (BMP‐2, 18 kDa), Cholesterol (386.65 g mol^−1^), Hydrogenated Soybean Phospholipid (HSPC, 762.09 g mol^−1^) were ordered from Macklin (Shanghai, China). Fibrin (340 kDa) and thrombin (37 kDa) were purchased from Ponsure Biological (Shanghai, China). Calcium acetate (158.166 g mol^−1^) was obtained from J&K Scientific (Beijing, China). Fetal bovine serum (FBS) and Penicillin‐Streptomycin solution (PS) were purchased from Gibco Invitrogen (Carlsbad, USA). BMP‐2 ELISA kit was purchased from Beyotime Biotechnology (Shanghai, China). PLGA (75:25, 70 kDa) was obtained from Titan (Shanghai, China). All the reagents and chemicals were purchased from commercial suppliers with analytical grade.

### Preparation of Liposome@PpIX@KGN@Diclofenac Sodium (LPKD)

The ROS responsive TK‐based liposome precursor (DSPE‐TK‐mPEG) was synthesized as previous work.^[^ ^23^ ^]^ To achieve a multifunction drug delivery, the responsive liposome nanoparticles was proposed and manufactured. Specifically, the Liposome@PpIX@KGN@Diclofenac sodium (LPKD) nanoparticles were manufactured by the thin film hydration and extrusion. Typically, the HSPC, Cholesterol, DSPE‐TK‐mPEG at a molar rate 85:10:5 was dissolved in chloroform (5 mL). Then, the sound sensitisers (PpIX) and Kartogenin (KGN) were dissolved in DMF (20 µL) and DMSO (40 µL) individually and added into the above mixture solution with mass 0.4% of the total liposome mass. The above mixture solution was evaporated at 50 °C by a rotary evaporator for 30 min to obtain phospholipid film and remove the high boiling point solvents (such as DMF, DMSO). Moreover, the film was dried overnight in a vacuum oven to ensure complete removal of the solvent. Next, 5 mL calcium acetate solution (120 mM) was added to the above‐mentioned flask and the solution was activated for 1 h at 65 °C. Then, the solution was extruded 30 times using a 200 nm polycarbonate membrane to obtain uniform size of nanoliposomes. The resulting nanoparticles were dialyzed using a dialysis membrane (MWCO = 7000 Da) with saline at 4 °C in a dark environment for 24 h to remove excess calcium acetate, HSPC, cholesterol, DSPE‐TK‐PEG, PpIX and KGN. Afterward, liposomes were incubated with diclofenac sodium (DS: liposomes, 20:1) for 10 min at 37 °C. To remove excess DS, the Gel column method (G50) was used, followed by 120 mM calcium acetate solution. All the nanoparticles (Lip, LPKD, LPB) were stored at ‐80 °C. Furthermore, the Liposome@PpIX@KGN (LPK) was synthesized using the same synthesis steps without DS.

### Preparation of Liposome@PpIX@BMP2 (LPB)

The Liposome@PpIX (LP) nanoparticle was synthesized using the method described above and then, the solution was mixed with BMP‐2 while stirring on an ice bath for 30 min. The solution was extruded 30 times through a polycarbonate membrane (200 nm). Subsequently, the Liposome@PpIX@BMP‐2 (LPB) nanoparticles were washed three times using ultrafiltration tubes (100 KDa) at 4 °C for 30 min at 4500 rpm.

### Preparation of Fibrin‐Based Nanoparticles Hydrogel (FT‐LPKD/LPB)

Fibrinogen precursor was prepared at a concentration of 100 mg mL^−1^ in saline at 37 °C and subjected to UV sterilization for 1 h. The thrombin precursor was adjusted to a concentration of 100 UI mL^−1^ using FLPKD solution (200 µg mL^−1^ KGN and 961.2 µg mL^−1^ DS). The FT‐LPKD hydrogel was fabricated using a double needle syringe at a volume ratio of 1:1 and solidified within 2 min at 37 °C. Similarly, the FT‐LPB hydrogel (200 ng mL^−1^ BMP‐2) was fabricated using the same method as described above.

### Preparation of Bi‐Layer PLGA Scaffolds

The PLGA scaffolds were fabricated via 3D cryogenic printing using a 40% PLGA solution in 1,4‐Dioxacyclohexane. The solution was extruded through a 21G dispensing needle at a speed of 2 mm s^−1^ and at a temperature of 25 °C. The lower‐layer scaffold was printed with a filament spacing of 500 µm and a height of 2 mm, while the upper‐layer scaffold was printed with a filament spacing of 300 µm and a height of 1 mm. The resulting bi‐layer PLGA scaffolds were cuboid‐shaped (35 × 35 × 3 mm) and dried using a lyophilizer for 3 days. All pre‐fabricated bio‐layer PLGA scaffolds were stored at −20 °C.

### Preparation of Multi‐Factors Loaded Nanohydrogels Composite Bilayer Scaffolds (PLGA‐FT‐LPKD/LPB)

PLGA‐FT‐LPKD/LPB composite scaffold was fabricated using the gelatin placeholder method. Specifically, the lower‐layer PLGA (35 × 35 × 2 mm) was infiltrated into 5% gelatin to occupy the pores using a silica gel ring with the height of 2 mm as a mold at 37 °C for 30 min, followed by storage at 4 °C for 10 min. Next, the FT‐LPKD hydrogel precursor was dropped into the upper‐layer PLGA scaffold for 2 min. The scaffolds were then placed in a 37 °C incubator to melt the gelatin (5 min) and washed thrice with DPBS at 37 °C. The lower‐layer PLGA scaffold was filled with the FT‐LPB hydrogel via the same procedure. The PLGA‐FT‐LPKD/LPB composite scaffols were sterilized using UV (Thermo Fisher Scientific, USA) with the wavelength of 253.7 nm under 90 µW cm^−2^ for 1 h and were subsequently used for cell and animal experiments. Specially, all nanohydrogel composite scaffolds with multi‐factors controlled release need to be used it right after it was ready.

### Transmission Electron Microscopy (TEM) Analysis

To analyze the morphology of the nanoparticles, LPKD nanoparticles solution containing 200 µg mL^−1^ KGN and LPB nanoparticles solution containing 200 ng mL^−1^ BMP‐2 were dropped onto the copper grid (ZhongJing Science Instrument, Beijing, Product ID: BZ11023A) and allowing them to dry. The TEM structural topography of the samples was then obtained using a JEMe1400 (120 kV, HITACHI, Japan).

### Dynamic Light Scatterometer (DLS) Analysis

The diameter and ζ‐potential values of LPKD and LPB nanocomposites were analyzed using a dynamic light scatterometer (DLS). Specifically, the Zetasizer Nano‐series (Nano ZS90, Malvern) was used to measure the diameter and ζ‐potential values. The stability of the nanoparticles was also evaluated by DLS at 37 °C for 7 days and their rupture was tested by DLS after 3 min of ultrasound (1 W cm^−2^) followed by 4 h of incubation.

### UV Spectra

To qualitatively analyze the drug‐loaded LPKD and LPB nanoparticles, the specific absorption peaks of various nanoparticles were detected by the UV–vis spectrophotometer in the range of 240–800 nm (Thermo Scientific, USA).

### Drug Loading

The concentrations of the KGN and DS loaded in FT‐LPKD hydrogels were quantified using HPLC at 270 and 278 nm (Shimadzu, 20 AT, Japan), respectively. Waters symmetrical C18 reversed‐phase column was performed to separation (250 mm × 4.6 mm, 5 µm) at a stable temperature of 40 °C. The mobile phase was composed of acetonitrile and 0.1% trifluoroacetic acid (TFA) in 50:50 (v:v) ratio to measured KGN at a flow rate of 2 mL min^−1^. The mobile phase consisted of methanol‐4% glacial acetic acid solution (70:30, v:v) was used to measured DS at a flow rate of 1 mL min^−1^ and a temperature of 30 °C. The loaded drug was collected by centrifugation after demulsification. The drug loading rate was determined via HPLC with the following equation:

(1) $$LE=\frac{M_{u}}{M_{t}}$$

wherein, the M*_t_* presents the total added amount of KGN and DS; the M*u* was the amount of loaded KGN and DS.

In addition, the loading rate of BMP‐2 for FT‐LPB hydrogels was measured using an ELISA kit.

### Drug Release Behavior

To evaluate the drug release behavior of PLGA‐FT‐LPKD/LPB scaffolds, all the samples were infiltrated into the tubes containing 5 mL DPBS and shaken at 120 rpm using a Bluepard shake bed (USA). All the samples were collected at 0.5, 2, 4, 8, 12, 24, 48, and 72 h to detect the release of DS using HPLC analysis with the parameters including Column: Phenomenex C18; Mobile phase: 50% acetonitrile and 50% 0.1% w/v trifluoroacetic acid; Flow rate: 2.0 mL·min^−1^ and then 200 µL of fresh DPBS was added to the tubes. KGN and BMP2 release behavior was evaluated by subjecting samples to ultrasound (1 W cm^−2^, 50% duty cycle) for 3 min on days 3, 5, and 11, followed by placement of the PLGA‐FT‐LPKD/LPB scaffolds into tubes containing 5 mL DPBS and shaking at 37 °C and 120 rpm. Samples were collected at 1, 2, 3, 5, 11, 14, 21, and 28 days and 200 µL DPBS was added to the medium after each collection. KGN concentration was quantified using HPLC with above‐mentioned same parameters. While BMP‐2 concentration was measured using ELISA kit according to the referred protocol (Beyotime biotechnology, Product ID PB045).

### SEM Characterization

The surface characterization of PLGA and PLGA‐FT‐LPKD/LPB scaffolds was performed by coating the surface of the samples with conductive Au using a high vacuum ion sputtering coater (ACE‐600) after lyophilization. The surface morphologies of the scaffolds were then observed using field emission scanning electron microscopy (JMF‐7500F, Japan).

### Rheological Behavior, Mechanical and Self‐Healing Properties

To evaluate the Rheological behavior of FT, FT‐LPB and FT‐LPKD hydrogels, the dynamic amplitude sweep, frequency sweep and strain sweep experiments were conducted with Rotational Rheometer (model Discovery HR‐20, USA). Moreover, the self‐healing property was confirmed using cut‐heal test. In addition, to evaluate the hydrogel effect on the mechanical properties of the scaffold, the compressive strength and compression modulus of the PLGA and PLGA‐FT‐LPKD/LPB scaffolds were measured by an electronic universal testing machine (INSTRON 5982, USA). The scaffolds were molded into cylindrical shapes (Ф5 mm × 3 mm) for testing.

### Swelling and Degradation Behavior of FT‐LPKD/LPB

The swelling and degradation ratios of FT‐LPKD/LPB hydrogels were determined using a gravimetric method. Dry samples (W_0_) were infiltrated into the 5 mL of DPBS and shaken at 120 rpm in 37 °C shake bed. All the swelling hydrogels samples were weighed (W_s_) after removing surface moisture at 1, 2, 4, 8, 24, and 48 h. The swelling ratio (E_s_) was calculated using the equation E_s_ = [(W_s_ – W_0_)/W_0_] × 100% (n = 4). The dry weight (W_t_) of freeze‐dried samples was measured at 1, 3, 5, 7, 14, and 28 days to evaluate the degradation ratio (D), which was calculated using the equation D = (W_0_ − W_t_)/W_0_ ×100% (n = 4).

### Cells Seeded in the PLGA‐FT‐LPKD/LPB

For in vitro 3D co‐culture of cells with PLGA‐FT‐LPKD/LPB scaffolds, BMSCs at passage 3 were extracted from the femoral marrow of SD rats (4 days) and seeded into the hydrogels. Specifically, BMSCs were suspended in a fibrinogen solution at a density of 2 × 10^7^ cells mL^−1^ and added to various groups, including PLGA‐FT, PLGA‐FT‐LPKD, PLGA‐FT‐LPB nanohydrogel scaffolds, with the size of diameter: 3 mm and height: 3 mm, which means there were 9.4 × 10^5^cells per 1 mm^3^ nanohydrogel scaffolds. These groups were prepared as described previously. The scaffolds were then co‐cultured with complete medium containing α‐MEM, 10% FBS and 1% PS at 37 °C and 5% CO_2_ for further study.

### CCK‐8 Assay and Proliferation

The cell viability of various scaffold groups was assessed by the CCK‐8 kit in vitro. Specifically, cells in PLGA‐FT‐LPKD/LPB scaffolds (Ф3.2 mm × 3 mm) were exposed to ultrasound (1 W cm^−2^, 50% duty cycle, 3 min) after 1, 3, and 7 days of culture. Following this, 150 µL of complete culture medium was added to 96‐well plates to incubate cells. The medium was then replaced by the fresh culture medium containing 10% CCK‐8 to co‐culture with cells for 2 h. The absorbance of the supernatant (100 µL) was analyzed via a multifunctional microplate reader (450 nm) to assess cell proliferation. To further investigate the cytotoxicity of the upper/lower‐layer scaffold system, the OD values of the FT, PLGA‐FT scaffolds, upper PLGA‐FT‐LPKD, lower PLGA‐FT‐LPB, upper PLGA‐FT‐LPKD+US and lower PLGA‐FT‐LPB+US groups were measured using the CCK‐8 kit on day 1.

### AM/PI Staining

After 3 days of 3D co‐culture, the viability of BMSCs in various scaffold groups was evaluated by the live/dead cell staining kit (Dojindo, Japan). Specifically, BMSCs were labeled by the propidium iodide (PI, 4 µM) and calcein‐AM (2 µM), which was incubated for 30 min in dark. All the samples were rinsed 3 times with PBS and visualized using confocal laser scanning microscopy (FV30‐ILSW, OLYMPUS, Japan). The excitation/emission wavelengths for calcein‐AM and PI were 490/515 nm and 530/580 nm, respectively.

### FITC/DAPI Staining

After 3 days of 3D co‐culture, the cytoskeleton of the BMSCs in various scaffold groups was assessed by FITC/DAPI staining. Specifically, BMSCs were labeled with FITC (5 µg mL^−1^) and DAPI (100 nM) for 1 h, followed by three washes with PBS. Then, all the samples were visualized by the confocal laser scanning microscopy (FV30‐ILSW, OLYMPUS, Japan), with FITC and DAPI exhibiting excitation/emission wavelengths of 488/530 nm and 359/454 nm, respectively.

### Migration of BMSCs

BMSCs (2 × 10^4^) were seeded onto silicone sheets (Ф2 mm × 5 mm) attached to the bottom of 48‐well plates. After 24 h, the silicone sheets were removed and the cells were co‐cultured with FT, PLGA‐FT, PLGA‐FT‐LPK/LPB, PLGA‐FT‐LPKD/LPB and PLGA‐FT‐LPKD/LPB +US in serum‐free medium for 12 and 24 h. The migration of the cells was evaluated using live staining with AM at 0, 12 and 24 h post‐co‐culture and images were captured using a microscope (Olympus, Japan). The area remaining without cells was measured using Image J to determine the migration rate.

### qRT‐PCR Analysis of BMSCs

The study analyzed the relative gene expression levels of BMSCs seeded in FT, PLGA‐FT scaffold, upper PLGA‐FT‐LPKD scaffold and lower PLGA‐FT‐LPB scaffold after 3D co‐culturing at different time points to differentiate toward cartilage and osteogenesis. For chondrogenic induction, the upper PLGA‐FT‐LPKD group in 6‐well plates after 24 h of incubation, the medium was replaced with chondrogenic induction medium for 14 days. Similarly, the medium of the lower PLGA‐FT‐LPB group was replaced with osteogenesis induction medium. Ultrasound was applied to the upper PLGA‐FT‐LPKD and lower PLGA‐FT‐LPB scaffold groups on days 3, 5, and 11. The scaffolds were homogenized and mRNA was extracted by the RNAiso plus kit (TaKaRa, Japan), followed by cDNA synthesis via the Reverse Transcription kit (Takara, Japan). The qRT‐PCR was performed by the TB Green qPCR (Takara, Japan) and the expression levels of cartilage‐related genes (ACAN, Sox9 and Col II) and osteogenesis‐related genes (RUNX2, Col II, BMP‐2, OCN) were tested using GAPDH as the internal control gene. The mRNA expression levels were calculated using the threshold cycle (Ct) method (R = 2^−ΔΔCt^).

### Staining Analysis of PLGA‐FT‐LPKD Nanohydrogel Composite Scaffolds In Vitro

Alcian blue staining was applied to evaluate the chondrogenic differentiation of BMSCs in FT, upper PLGA‐FT scaffold, upper PLGA‐FT‐LPKD scaffold and upper PLGA‐FT‐LPKD scaffold with ultrasound treatment at days 3 and 5 in chondrogenic induction medium. All complex scaffolds were fixed by 4% paraformaldehyde for 30 min, washing with DPBS until no color remained in DPBS. Then, the 3D images of the Alcian blue staining were obtained using a bodyscope and microscope. Additionally, the upper PLGA‐FT‐LPK scaffold and upper PLGA‐FT‐LPKD +US scaffold were also used in chondroblast differentiation as described above.

To assess the differentiation of BMSCs toward osteogenesis, the alkaline phosphatase (ALP) activity was measured in FT, PLGA‐FT scaffold, lower PLGA‐FT‐LPB scaffold and lower PLGA‐FT‐LPB scaffold with ultrasound treatment at day 3 and 5 in osteogenesis induction medium. The ALP staining was performed for 1 h after fixation with 4% paraformaldehyde. The images were captured as previously described. After 7 days of culture, the scaffolds were frozen using liquid nitrogen and homogenized. The cells were then lysed by RIPA lysis buffer and the proteins were collected through centrifugation at 1200 rpm/min. The Alkaline Phosphatase assay kit (Beyotime, China) was used to measure the ALP activity.

To assess the differentiation of BMSCs to osteogenesis in FT, PLGA‐FT scaffold, lower PLGA‐FT‐LPB and lower PLGA‐FT‐LPB+US with ultrasound treatment at day 3, 5, and 11 in osteogenesis induction medium, alizarin red staining was used. After incubation with alizarin red for 1 h at room temperature, the scaffolds were washed with DPBS and then incubated overnight with 5% Sodium dodecyl sulfate (SDS, Sigma‐Aldrich, USA). The solution absorbance was measured at 405 nm (microplate reader) for quantitative analysis.

### Animal Procedure

All the female SD rats were obtained from Changzhou Cavens Laboratory Animal Co., Ltd (6 W, 180–200 g). Following general anesthesia induced by intraperitoneal injection of pentobarbital sodium (35 mg kg^−1^), the osteochondral defect model (Ф3.2 × 3 mm) was created in the center of the patellar groove of the femurs by an electric drill. Then, the control, PLGA, PLGA+FT, PLGA‐FT‐LPK/LPB, PLGA‐FT‐LPKD/LPB and PLGA‐FT‐LPKD/LPB+US scaffolds were implanted into the femur defects. The PLGA‐FT‐LPK/LPB+US and PLGA‐FT‐LPKD/LPB+US groups were exposed to supersonic waves (1 W cm^−2^, 3 min) on 3, 5, and 11days. All the SD rats were euthanized with pentobarbital and their femurs were collected at 4 W and 8 W post‐treatment. All the experimental protocols were approved by the Ethics Committee of Shanghai University (No. ECSHU2024‐121).

### Micro‐CT Evaluation

All the SD rats femurs were fixed in 4% formalin for two days and subsequently imaged using a Micro‐CT scanner (Inveon PET/CT, Siemens, Germany). The cylindrical region of interest (ROI) was concentrically positioned over the modeling site with a diameter of 3.2 mm and a height of 3 mm. The 3D reconstruction was performed by the computer‐assisted design software (Midivi, Jinse Medical, Changzhou, China). Moreover, the BV/TV and Tb.Th data were calculated over the ROI.

### Histological and Immunohistochemical Analysis

The SD rats femurs were decalcified in a decalcification solution (Servicebio, Shanghai) for 4 W. After dehydration with graded ethanol, All the samples were paraffin‐embedded in desirable location (regeneration tissue location) and sliced into 10 mm sections. The femur slices from different groups were stained with safranin hematoxylin‐eosin (H&E), O/fast green (S‐F) and Masson staining (Servicebio, Shanghai). The special expression markers of IL‐1*β*, COL II and COL I were analyzed by the immunohistochemical staining in desirable area. Moreover, the staining images were captured using an inverted microscope (Olympus, Japan). In addition, the major organs were evaluated for safety by pathological staining (H&E) after 8 W of treatment (e.g., heart, liver, spleen, lung and kidney).

### Statistical Analysis

All the data were analyzed using mean ± standard deviation (SD) from more than three samples. All the statistical analysis were performed by the GraphPad Prism software. The one‐way or two‐way analysis of variance (ANOVA) was used to determine significant differences groups. Differences with a P ≤ 0.05 were considered statistically significant.

## Conflict of Interest

The authors declare no conflict of interest.

## Supporting information



Supporting Information

## Data Availability

The data that support the findings of this study are available from the corresponding author upon reasonable request.
