# Supplementary material for: ROS‐Activated Nanohydrogel Scaffolds with Multi‐Factors Controlled Release for Targeted Dual‐Lineage Repair of Osteochondral Defects
Source: Adv Sci (Weinh). 2025 Mar 29;12(20):2412410. doi: 10.1002/advs.202412410 (PMC12120736; doi:10.1002/advs.202412410)
Supplement: Supplementary file 1 — Supporting Information [file ADVS-12-2412410-s001.docx]

**Supporting information**

**ROS-activated Nanohydrogel Scaffolds with Multi-factors Controlled Release for Targeted Dual-Lineage Repair of Osteochondral Defects**

Xiuhui Wang, Shunli Wu, Ruiyang Li, Huijian Yang, Yue Sun, Zijie Cao, Xiao Chen, Yan Hu, Hao Zhang, Zhen Geng, Long Bai, Zhongmin Shi*, Ke Xu*, Hongbo Tan*, Jiacan Su*

X. Wang, S. Wu, Z. Geng, Y. Sun, L. Bai, K. Xu, J. Su

Institute of Translational Medicine, Shanghai University, Shanghai 200444, China

Organoid Research Center, Shanghai University, Shanghai, 200444, China

National Center for Translational Medicine (Shanghai) SHU Branch, Shanghai University, Shanghai, 200444, China

R. Li, X. Chen, Y. Hu, H. Zhang, J. Su

Department of Orthopedics, Xinhua Hospital, Shanghai Jiao Tong University School of Medicine, Shanghai, 200092, China

H. Yang

Department of Clinical Laboratory, Shanghai Zhongye Hospital, Shanghai, 200941, China

Z. Cao, H. Tan

Department of Orthopaedics, People’s Liberation Army Joint Logistic Support Force 920th Hospital, Kunming, 650118, China

Z. Shi

National Center for Orthopaedics, Department of Orthopedic Surgery, Shanghai Sixth People’s Hospital, Shanghai 200233, China

E-mail: [szm1972@sjtu.edu.cn](mailto:szm1972@sjtu.edu.cn) (Z. Shi), , [kexu@shu.edu.cn](mailto:kexu@shu.edu.cn) (K. Xu), [tantoo@163.com](mailto:tantoo@163.com) (H. Tan), [jiacansu@shu.edu.cn](mailto:jiacansu@shu.edu.cn) (J. Su)


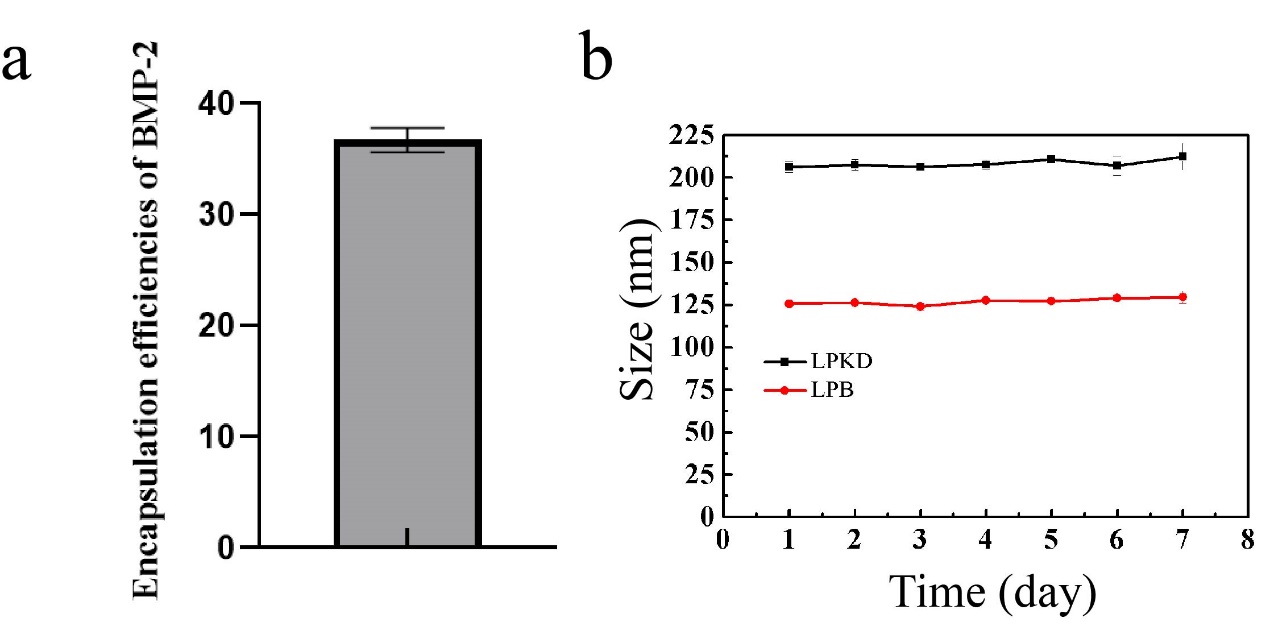


**Figure S1.** (a) Encapsulation efficiencies of BMP-2 by ELISA Kit. (b) Hydrodynamic sizes of the LPB and LPKD nanoparticle stored at 37 °C for 7 days.


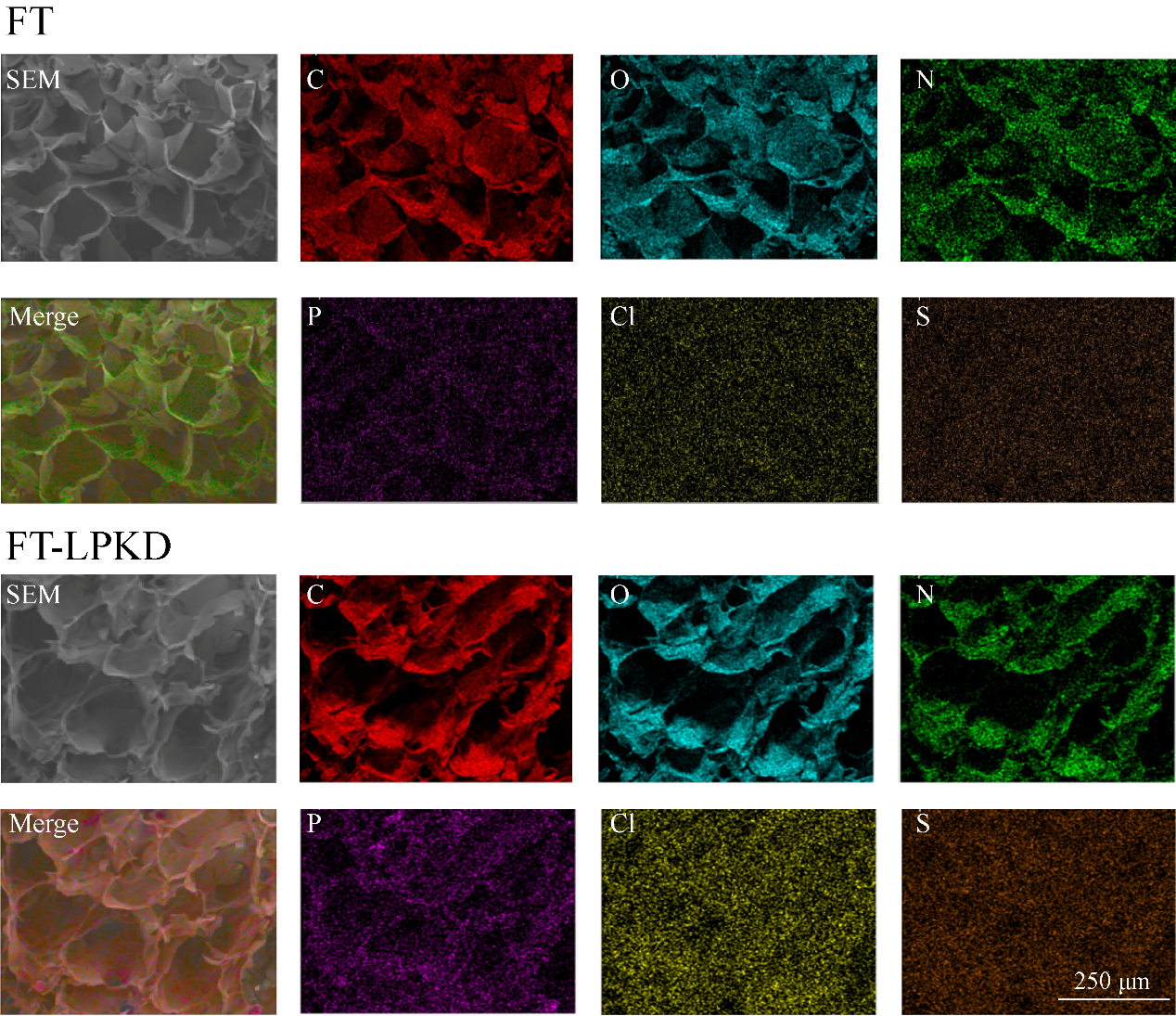


**Figure S2.** SEM-EDX mapping of C, O, N, P, Cl and S elements in FT hydorgels and FT-LPKD nanocomposite hydrogels.


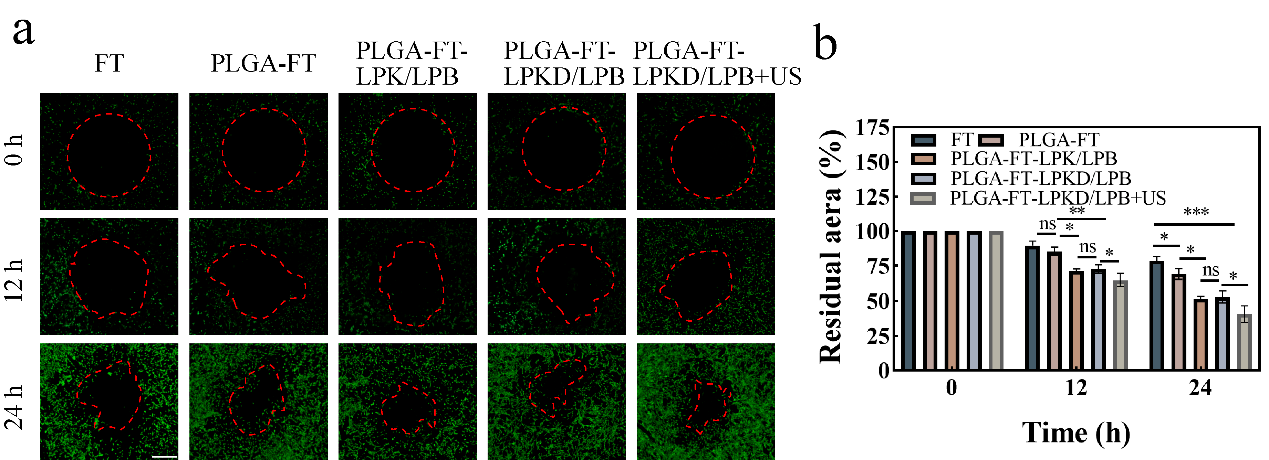


**Figure S3.** (a) Migration of BMSCs via co-culture with hydrogel composite scaffolds was assessed by AM staining at 0, 12 and 24 h (Scale bar: 500 μm). (b) Quantitative analysis of the remaining area was performed using ImageJ.


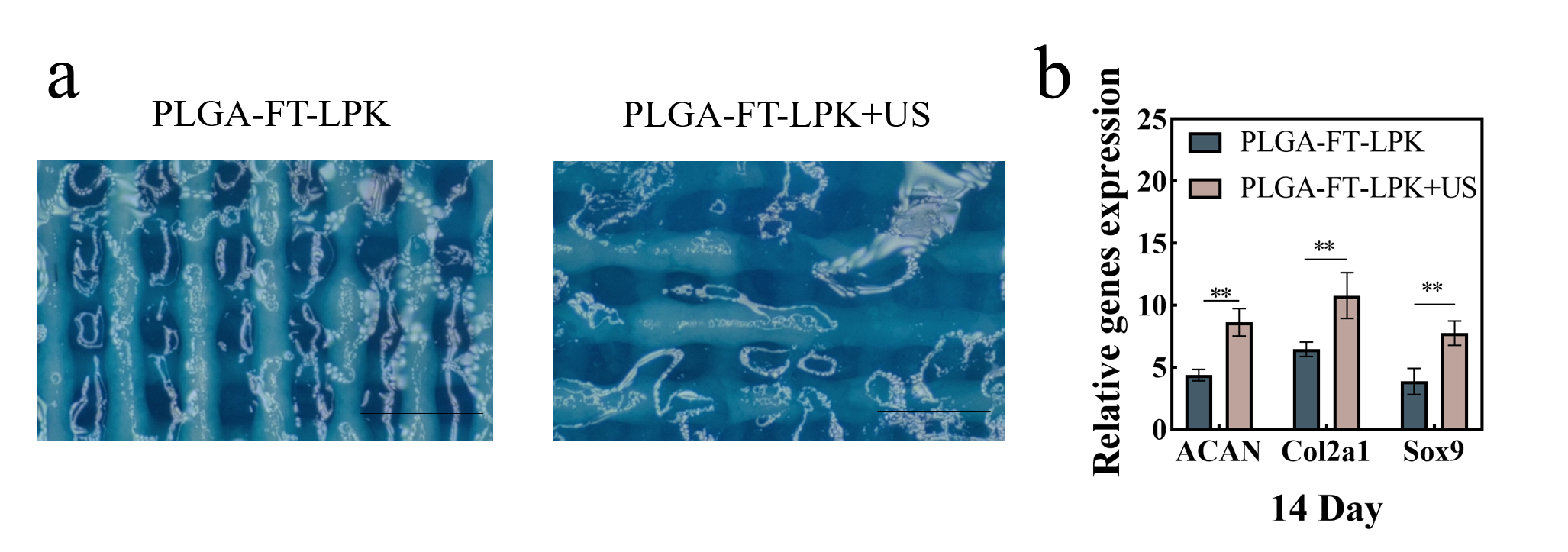


**Figure S4.** Assessment of chondrogenic differentiation capacity of PLGA-FT-LPK hydrogel composite scaffolds. (a) Alcian blue staining images after being cultured for 7 days (Scale bar: 1000 μm). (b) The relative expression of typical chondrogenic gene markers after being cultured for 14 days.


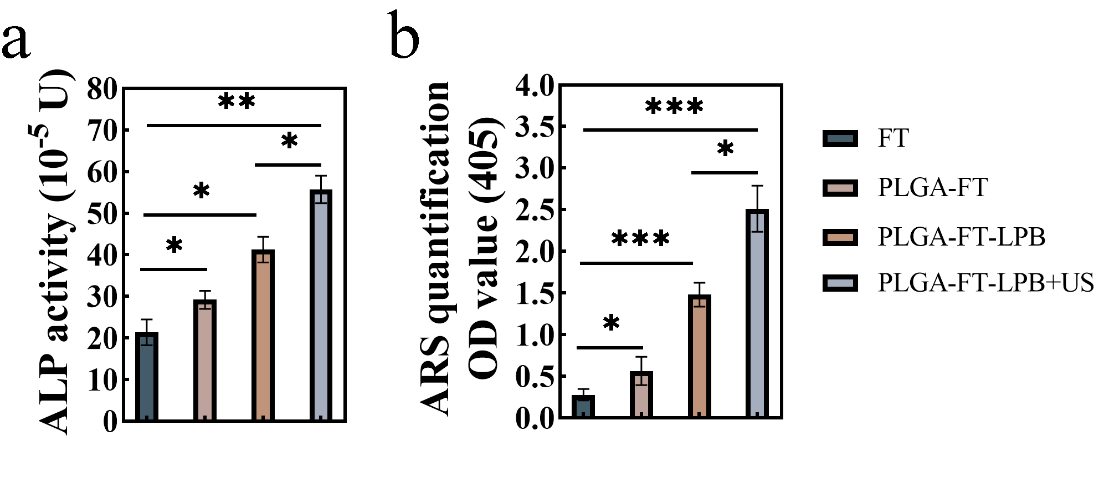


**Figure S5.** Quantitative analysis of ALP and ARS staining. (a) ALP activity. (b) ARS quantification value.


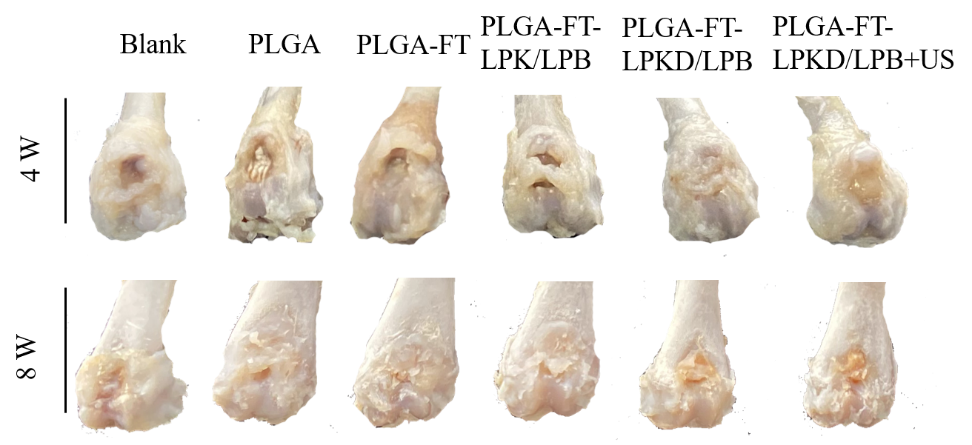


**Figure S6.** Gross observation of knee after implanted hydrogel composite scaffolds for 4 and 8 weeks.


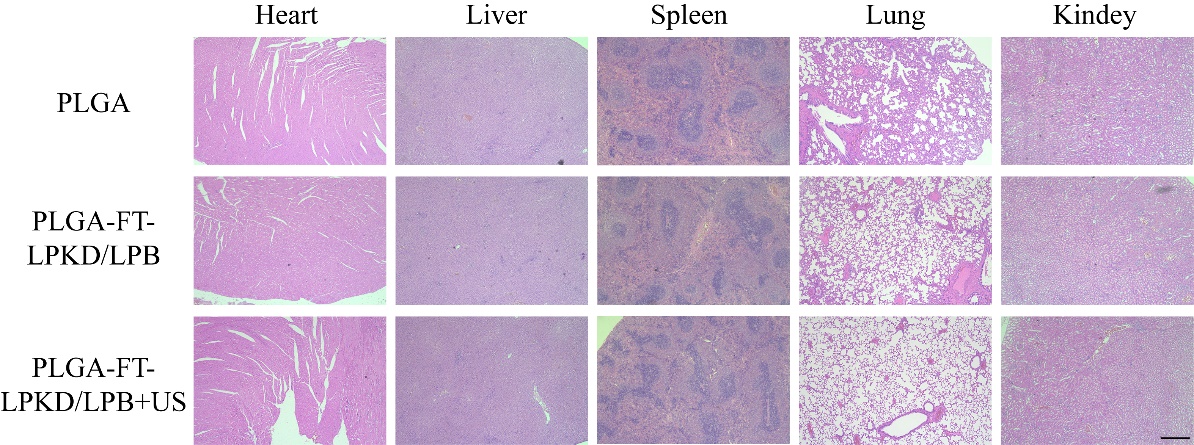


**Figure S7.** H&E histology analysis of heart, liver, spleen, lung and kidney in rats after 8 weeks in different groups. (Scale bar: 500 μm)


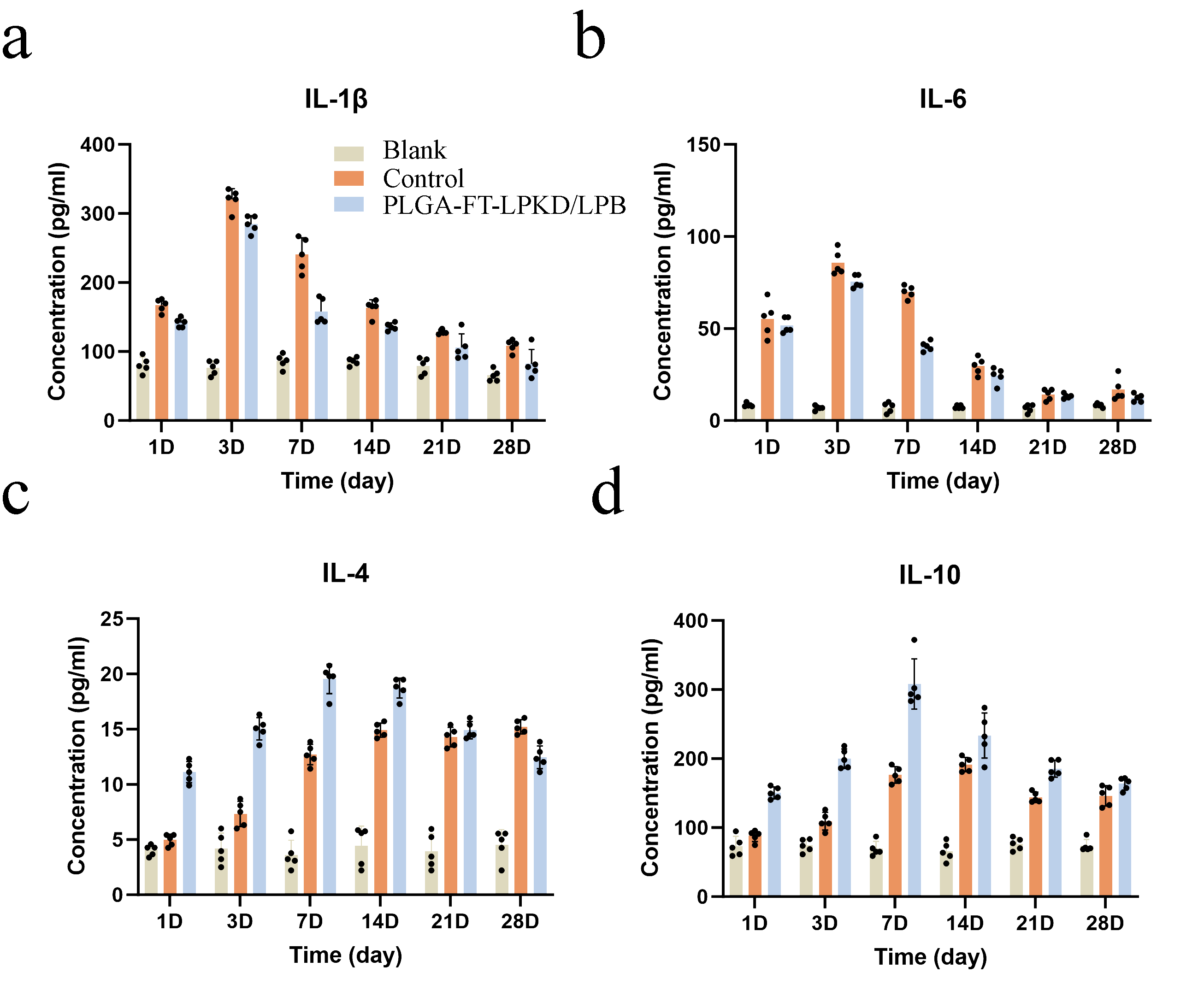


**Figure S8.** Time-course expression of cytokines including IL-1β, IL-6, IL-4 and IL-10 after

being implantation of PLGA-FT-LPKD/LPB nanohydrogel scaffolds for 1, 3, 7, 14, 21 and 28 days *in vivo*.
